# Nanofluid Heat Transfer: Enhancement of the Heat Transfer Coefficient inside Microchannels

**DOI:** 10.3390/nano12040615

**Published:** 2022-02-11

**Authors:** Kevin Apmann, Ryan Fulmer, Branden Scherer, Sawyer Good, Jake Wohld, Saeid Vafaei

**Affiliations:** Mechanical Engineering Department, Bradley University, Peoria, IL 61606, USA; kapmann@mail.bradley.edu (K.A.); rfulmer@mail.bradley.edu (R.F.); bscherer@mail.bradley.edu (B.S.); sgood@mail.bradley.edu (S.G.); jwohld@mail.bradley.edu (J.W.)

**Keywords:** nanoparticles, microchannels, connector, heat transfer coefficient, thermal conductivity and viscosity

## Abstract

The purpose of this paper is to investigate the effects of a connector between two microchannels, for the first time. A brief literature review is provided to offer a better understanding on the impacts of concentration and the characteristics of nanoparticles on thermal conductivity, viscosity, and, consequently, the heat transfer coefficient inside the microchannels. The given literature review aims to help engineer nanofluids to enhance the heat transfer coefficient inside the microchannels. In this research, Fe_3_O_4_ nanoparticles were introduced into the base liquid to enhance the heat transfer coefficient inside the microchannels and to provide a better understanding of the impact of the connector between two microchannels. It was observed that the connector has a significant impact on enhancing the heat transfer coefficient inside the second microchannel, by increasing the level of randomness of molecules and particles prior to entering the second channel. The connector would act to refresh the memory of the fluid before entering the second channel, and as a result, the heat transfer coefficient in the second channel would start at a maximum value. Therefore, the overall heat transfer coefficient in both microchannels would increase for given conditions. The impacts of the Reynolds number and introducing nanoparticles in the base liquid on effects induced by the connector were investigated, suggesting that both factors play a significant role on the connector’s impact on the heat transfer coefficient.

## 1. Introduction

The thermal management of systems plays an increasingly important role in modern society. As devices become more complex, especially electronics, it becomes necessary to have even more effective thermal management systems. Since space and money are often limited for these systems, they must be made as simple, effective, and small as possible. Since the smaller and lighter heat exchangers have been in demand for aviation technology, the research on nanofluid forced convection heat transfer is becoming more prevalent to achieve a more compact design. The light, miniaturized, and efficient thermal management play a significant role in space technology and NASA programs. Much research has been directed to finding more effective and efficient methods to manage heat without increasing the size of the cooling system. One way to achieve more effective thermal management is by enhancing the thermal properties of the fluid used in the system. In 1995, Choi and Eastman [1] suggested adding nanoparticles to fluids to enhance the heat transfer abilities of these base liquids [1]. It was postulated that, as metals have significantly higher thermal conductivity than common base liquids, suspending metallic nanoparticles in fluids used for thermal management would greatly increase their thermal conductivity and, thus, their effectiveness as coolants. Since then, various different nanoparticles have been studied, in conjunction with a variety of base liquids. Metal oxides were initially suggested by Choi and Eastman [1], as being one of the most commonly studied nanofluid types, but non-metallic, carbon-based, ceramic, and oxide-derived compounds have all been studied [2,3,4,5,6]. Varying the type, concentration, and even shape of the nanoparticles, along with the base liquid, can shift the fluid properties, and most importantly its heat transfer coefficient. Nanofluids with an increased heat transfer coefficient are much more effective cooling agents in a variety of situations. Moreira et al. [7] proposes that this could be useful in applications where space is limited, such as electronics and solar cells. Applications such as electronics, along with high-powered engines and other scenarios where there are high amounts of generated heat, are also areas where Chabi et al. [3] suggest that nanoparticles could be used to increase the heat transfer coefficient of the cooling liquids in use. This would allow for faster and more effective cooling of these systems.

Any addition to a base liquid can alter the overall fluid properties of the resulting mixture. The addition of nanoparticles often has a profound effect on the base liquids’ thermal conductivity. Choi and Eastman’s early work with copper nanoparticles [1] linked the fluid’s overall thermal conductivity with the volume fraction of the nanoparticles in the base liquid. Chiam et al. [8] also produced results which link an increased volume concentration of a nanoparticle to a higher thermal conductivity. Another factor affecting thermal conductivity was found to be size. Pryazhnikov et al. [9] found that larger nanoparticles were found to increase the thermal conductivity of the fluid more than smaller nanoparticles. They also determined that the thermal conductivity of a nanofluid was dependent on the material of the nanoparticles. The addition of a surfactant to a nanofluid did not produce a linear change in thermal conductivity. Rather, it was found that a surfactant increased the thermal conductivity up to a certain optimal point, after which additional surfactant caused the nanofluid thermal conductivity to drop [10]. An additional factor in the thermal conductivity is the base liquid used for creating the nanofluid. In certain cases, the nanofluid properties will follow similar enhancement trends to the underlying base fluid for different fluid flow conditions [11].

The viscosity of any nanofluid has its starting point in the properties of its base liquid. These properties are then subsequently altered as nanoparticles are introduced. Lee et al. [12] investigated changes in density for low volume concentrations of nanoparticles, which showed that increasing the volume fraction of the nanoparticles would slightly increase the nanofluids viscosity. It was also found that the viscosity for low concentrations of the nanoparticles did not follow a linear relationship, as was predicted by current models. Viscosity increases with higher nanoparticle concentration, but will decrease greatly with temperature, even for high nanoparticle concentrations [8]. In a comprehensive review of nanoparticle literature, Nadooshan et al. [13] found that nanofluids with a low volume fraction of nanoparticles typically demonstrated Newtonian fluid behavior. Correspondingly, at high volume fractions, nanofluids were more likely to exhibit non-Newtonian behavior and, thus, have viscosities dependent on the shear conditions of the fluid flow. Garoosi [14] found that the base liquid of a nanofluid played a role in influencing the viscosity enhancement ratio and presented equations which could be used to calculate the dynamic viscosities of water, glycerol, ethanol, ethylene glycol, and propylene glycol. These five liquids represent some of the most commonly used base liquids in nanofluid studies.

There are many possible applications of nanofluids, each with various different design requirements. Thus, it is important to use an optimized nanofluid for any particular application. The nanoparticles and base liquid must be properly selected based on the needed viscosity, thermal conductivity, and heat transfer coefficient for the specific thermal management system being designed. In order to increase the heat transfer coefficient, it is beneficial to increase the thermal conductivity and decrease the viscosity of the nanofluids. Since the properties of the base fluid significantly affect the nanofluid properties, base fluids with higher thermal conductivity and lower viscosity, such as water, are desirable for optimizing a nanofluid [15]. The effects of additional parameters, such as possible surfactant and the nanoparticle characteristics on thermal conductivity and viscosity, have been less definitively determined. Therefore, additional research is needed to determine the combined effect of these parameters to optimize the nanofluids. Some surfactants for example can increase or decrease thermal conductivity depending on the concentration of the surfactant [10]. In order to create the optimum nanofluid, the effect of all parameters on both thermal conductivity and viscosity need to be considered.

The addition of nanoparticles has a profound impact on the heat transfer coefficient of the base liquid they are added to. Even small amounts of nanoparticles relative to the volume of base fluid can raise the thermal conductivity of the fluid according to Hussien et al. [6]. Akhavan-Zanjani et al. found that graphene in amounts as low as 0.02% volume concentration would enhance the heat transfer coefficient of water by over 10% [16]. Godson et al. [17], after conducting a review of published nanofluid literature, concluded that nanofluids consistently exhibit higher heat transfer coefficients than their base fluids. They also noted that, at the time of publishing, there were few models that could predict the heat transfer behavior in nanofluids and practically none included effects, such as particle size or shape, among other parameters. Xie et al. [18] experimentally worked with Al_2_O_3_, ZnO, TiO_2_, and MgO nanofluids and also found that heat transfer coefficient increased across the board over the base fluids. They proposed a model for calculating the convective heat transfer coefficient which considers the fluids specific heat and other external factors.

While many studies have dealt with the heat transfer coefficient in a single continuous microchannel, few have dealt with the effect of gaps arising from the joining of two microchannels. Gaps such as these both commonly arise in applications and have the potential to shift the heat transfer properties of the fluid. The purpose of this paper is to experimentally examine the effects of a non-heated gap region on the heat transfer coefficient of nanofluids in a series of microchannels. This experimental data will be analyzed to determine the possible cause of any novel behavior and compared with experimental and theoretical models. This paper also includes a review of current and relevant literature on the effects of nanoparticles on a fluid’s thermal conductivity, viscosity, and heat transfer coefficient.

## 2. Review Study of the Heat Transfer Coefficient and the Effects of Concentration and Characteristics of Nanofluids on Thermal Conductivity

The addition of nanoparticles typically modifies the preexisting thermal properties of base liquids, including thermal conductivity and viscosity. Both parameters have a great potential to modify the heat transfer coefficient inside the channels. In general, the heat transfer coefficient can be affected by the thermal conductivity, viscosity, and flow regime of the fluid.

### 2.1. Variation in the Thermal Conductivity of Various Base Fluids with Temperature

**Figure 1:**
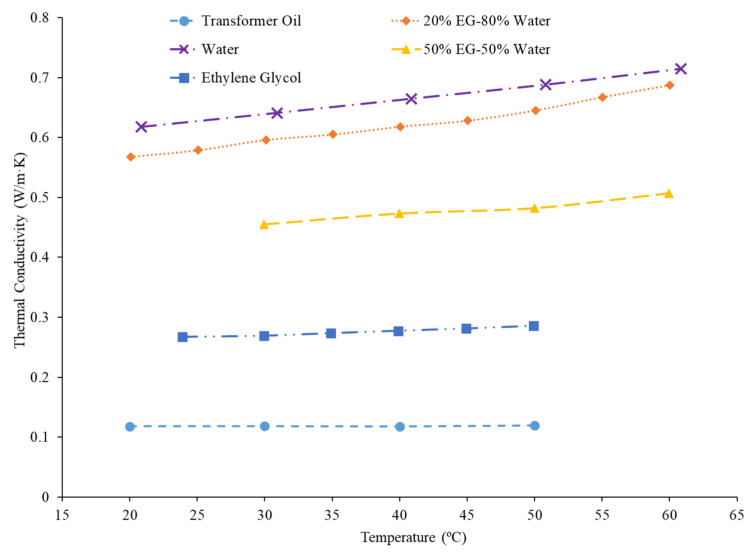
Thermal conductivity of Al_2_O_3_ nanofluids at a volume fraction of 1% with various base fluids: 50% EG–50% Water [8], Transformer Oil [19], 20% EG–80% Water [21], Water [22], Ethylene Glycol [23].

There are several different base liquids that are commonly used in the creation of nanofluids. The most common base liquids are water, ethylene glycol, and water–ethylene glycol mixtures. Several other base liquids frequently used are propylene glycol, kerosene, and various oils. Generally, the thermal conductivity of the nanofluid is highly dependent on the thermal conductivity of the base liquid, and base liquids with a higher thermal conductivity will produce nanofluids with a higher thermal conductivity [8,19,20,21]. Patel et al. [19] measured the thermal conductivity of nanofluids in various base liquids such as water, ethylene glycol, and transformer oil. There was a clear relationship between the thermal conductivity of the base fluid and the nanofluid. The highest thermal conductivity base liquid used was water, and the water-based nanofluids had the highest thermal conductivity as well. Additionally, transformer oil had the lowest thermal conductivity of the base liquids used and the transformers oil nanofluids had the lowest thermal conductivity. Commonly, water and ethylene glycol will be used in a mixture for the creation of nanofluids. Chiam et al. [8] studied the thermal conductivity of Al_2_O_3_ nanofluids with base liquids, i.e., water–ethylene glycol mixtures with mixture ratios of 60%:40%, 50:50%, and 40%:60% ethylene glycol:water. The thermal conductivity increased with greater concentrations of water due to the higher thermal conductivity of water compared to ethylene glycol. Since water has a higher thermal conductivity, the mixtures’ thermal conductivity increases with an increase in water concentration. Sundar et al. [15] also studied the thermal conductivity of nano-diamond (ND) nanofluids with base liquids that were water–propylene glycol mixtures with mixture ratios of 20%:80%, 40%:60%, and 60%:40% propylene glycol:water. As seen with the ethylene glycol–water mixtures, the thermal conductivity increased with a greater concentration of water due to the high thermal conductivity of water compared to propylene glycol. Figure 1 demonstrates the effect of various base fluids for Al_2_O_3_ nanofluids at a volume fraction of 1%. The water-based nanofluid has the highest thermal conductivity, while the transformer oil-based nanofluid has the lowest thermal conductivity. The transformer oil-based nanofluid was almost constant as the temperature increased while the other nanofluids demonstrated an increase in thermal conductivity as the temperature increased. Of the base fluids chosen, transformer oil is the only one to decrease in thermal conductivity as temperature increases. In a nanofluid, the increase in temperature will create more motion of the nanoparticles, leading to more energy transfer [20]. As a result, nanoparticles will lead to greater increase in thermal conductivity at higher temperatures. It is possible that, in the transformer oil nanofluid, these two effects counteract each other. The higher temperatures reduce the thermal conductivity of the base fluid, but the energy transfer in the nanoparticles increases [20]. As a result, the thermal conductivity is relatively constant. For the ethylene glycol–water mixtures, as the concentration of water increases, the thermal conductivity of the nanofluid will increase. Therefore, the thermal conductivity of a nanofluid can be generally predicted based on the thermal conductivity of the base fluid. Higher thermal conductivity base fluids will produce higher thermal conductivity nanofluids. Water is the highest thermal conductivity base fluid that is commonly used in nanofluids. In fluid mixtures, a higher concentration of water leads to higher thermal conductivity.

### 2.2. Effects of Nanoparticle Material on Thermal Conductivity

**Figure 2:**
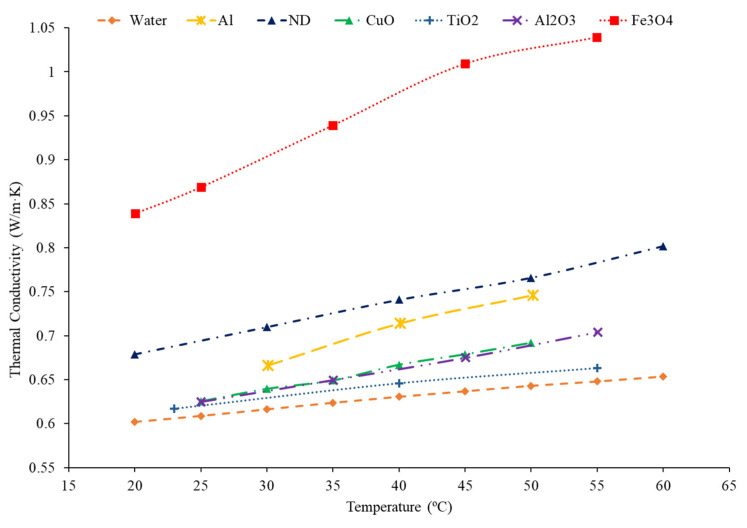
Thermal conductivity of water-based nanofluids at a volume fraction of 1% made with various nanoparticles: Al [19], CuO [27], TiO_2_ [28], ND [29], Al_2_O_3_ [30], Fe_3_O_4_ [31].

There are many materials that can be used to create nanofluids such as metal, metal oxides, nano-diamonds, and various carbon nanotubes. Although it has been proposed that the thermal conductivity of the nanoparticle will have a correlation to the thermal conductivity of the nanofluid, that relationship has not been clearly established. Gangadevi et al. [24] studied the thermal conductivity of Al_2_O_3_–water and CuO–water nanofluids. Both nanofluids demonstrated that, as the temperature of the nanofluids increased, there was an increase in thermal conductivity as well. The enhancement in the CuO–water nanofluid was greater than the enhancement in the Al_2_O_3_–water nanofluid. The average increase in the Al_2_O_3_–water nanofluid thermal conductivity was 11.23%, while the CuO–water had a 12.15% average increase in thermal conductivity, with both nanofluids at a volume fraction of 0.2% and across a temperature range of 20 °C to 60 °C. CuO nanoparticles have been found to have a higher thermal conductivity than Al_2_O_3_ nanoparticles, which could explain the higher thermal conductivity of the CuO nanofluid compared to the Al_2_O_3_ base fluid. Xing et al. [25] measured the thermal conductivity of water-based nanofluids containing short single-wall carbon nanotubes (S-SWCNT), long single-wall carbon nanotubes (L-SWCNT), and multiwall carbon nanotubes (MWCNT). At a temperature of 60 °C and a volume fraction of 0.24%, the L-SWCNT–water nanofluid had a thermal conductivity enhancement of 9.8%, while the S-SWCNT–water and MWCNT–water nanofluids had thermal conductivity enhancements of 5.07% and 3.38%, respectively. The largest increase in thermal conductivity being in the L-SWCNT–water nanofluid could be caused by the high aspect ratio of the L-SWCNT particles. The high aspect ratio allows for greater energy transfer between particles since there will be greater contact between the particles. Sridhar et al. [26] measured the thermal conductivity of Ag–water and SnO_2_–water nanofluids at volume fractions between 0.05% and 0.15%. Both nanofluids increased in thermal conductivity as the concentration increased and the Ag–water nanofluid thermal conductivity was higher than the SnO_2_–water nanofluid thermal conductivity. Ag nanoparticles also have a higher thermal conductivity than the SnO_2_ nanoparticles. The high thermal conductivity of the particles could contribute to the high overall nanofluid thermal conductivity. This suggests a logical conclusion that a higher thermal conductivity nanoparticle will lead to a higher thermal conductivity. Pryazhnikov et al. [9] studied the thermal conductivity of nanofluids with a water base liquid and nanoparticle materials of SiO_2_, TiO_2_, ZrO_2_, Al_2_O_3_, and CuO. At a volume fraction of 2%, there was no direct correlation between the thermal conductivity of the nanofluid and the thermal conductivity of the nanoparticles. Therefore, although there is evidence that higher thermal conductivity of nanoparticles will lead to a higher thermal conductivity of nanofluid, there are other parameters that must be considered. In Figure 2, the thermal conductivity of water-based nanofluids at a volume fraction of 1% and various nanoparticle materials is shown. The Fe_3_O_4_ nanofluid has the highest thermal conductivity followed by the nano-diamond nanofluid. Nano-diamonds have a higher thermal conductivity than Fe_3_O_4_ suggesting that nanoparticle thermal conductivity does not necessarily predict nanofluid thermal conductivity. The TiO_2_ nanofluid has the lowest thermal conductivity and TiO_2_ nanoparticles have the lowest thermal conductivity of the particles considered. There may be some correlation between nanoparticle thermal conductivity and nanofluid thermal conductivity, but there are exceptions. In some cases, increasing the thermal conductivity of the nanoparticles will lead to a higher overall thermal conductivity of the nanofluid. There are exceptions in some cases where nanoparticles with lower thermal conductivity will produce nanofluids with high thermal conductivity.

### 2.3. Effects of Nanoparticle Concentration on Thermal Conductivity

**Figure 3:**
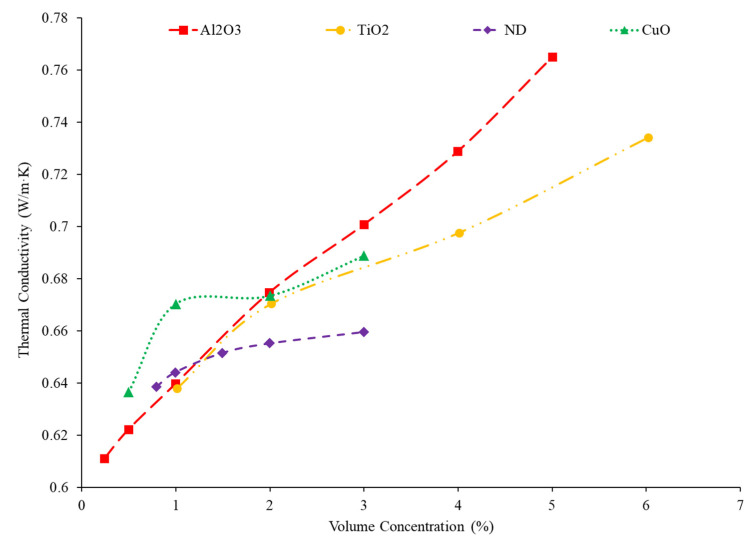
Thermal conductivity as a function of volume fraction for several water-based nanofluids at a constant temperature: CuO [19], TiO_2_ [9], ND [32], Al_2_O_3_ [35].

Many researchers have found that as the concentration of nanoparticles in a nanofluid increases, the thermal conductivity of the nanofluid increases as well. Yeganeh et al. [32] measured the thermal conductivity of nano-diamond (ND)–water nanofluids at volume concentrations ranging from 0.8% up to 3%. Over the span of temperatures of 30 °C to 50 °C, with the increase in concentration of the nanofluid, the thermal conductivity increased with a nonlinear relationship. Sundar et al. [33] studied the relationship of nanoparticle concentration on the thermal conductivity of Fe_3_O_4_–water nanofluids. There was an increase in thermal conductivity as the volume fraction increased from 0.2% up to 2%. The Brownian motion of the particles also increased as the concentration increased, which could be the cause of the increase in thermal conductivity of the nanofluid. Micro-convection occurs in the surrounding liquid molecules as a result of the Brownian motion, which could also cause an increase in thermal conductivity. Godson et al. [34] studied the effect of concentration on the thermal conductivity of Ag–water nanofluids. The volume fractions tested were 0.3%, 0.6%, and 0.9%. As the volume fraction on the nanoparticles increased, the thermal conductivity of the nanofluids increased as well. The thermophoresis of the nanoparticles and Brownian velocity of the particles also increased as the volume fraction increased. Thermophoresis is the motion of particles created by a temperature gradient within the fluid. In some cases, the effect of thermophoresis could be greater than the effect of Brownian motion. Both of these factors would contribute to increased particle collision. Generally, these particle collisions increase the energy transfer between particles which would increase the thermal conductivity of the nanofluid. Figure 3 gives the thermal conductivity of several water-based nanofluids at a constant temperature as the volume fraction increases. For each of the nanofluids, as the concentration increases, the thermal conductivity increases as well. Additionally, the rate of increase in thermal conductivity is generally greatest in lower concentrations. At higher concentrations, the nanoparticles are more likely to agglomerate, potentially reducing the further enhancement at higher concentrations. Increasing the concentration in a nanofluid will increase the thermal conductivity from an increase in Brownian motion and particle interactions.

### 2.4. Effects of Nanoparticle Shape on Thermal Conductivity

Frequently, nanoparticles will have a spherical shape, but there are other shapes, such as cylindrical, cubic, wire, or needle shapes, that have been studied. The nanoparticle shape is less frequently studied than other nanoparticle shapes, but nonetheless has an impact on the thermal conductivity. Generally, nanoparticle shapes with higher surface area to volume ratios will create nanofluids with a greater thermal conductivity. Maheshwary et al. [36] studied the thermal conductivity of water-based nanofluids containing cubic, rod, and spherical TiO_2_ nanoparticles. The nanofluids created using cubic-shaped nanoparticles had the highest thermal conductivity, which could be because of the three shapes tested and the fact that the cubic nanoparticles had the highest surface area to volume ratio. The advantage of an increased surface area to volume ratio is that heat transfer is a function of the surface area; therefore, a greater surface area in the nanoparticle will facilitate a higher heat transfer. Cubic nanoparticles will not necessarily always have the highest surface area to volume ratio, as depending on the particular dimensions of the nanoparticles, it is possible for other shapes to have a higher surface area to volume ratio. Main et al. [37] measured the thermal conductivity of ionic liquid based nanofluids, specifically Al_2_O_3_-1-Butyl-3-methylimidazolium bis(trifluoromethylsulfonyl) imide ([C4mim] [NTf2]) nanofluids, with various nanoparticle shapes. The nanoparticles used had a variety of shapes, such as sphere, rod, and needle. Of the nanofluids tested, the highest thermal conductivity was in the nanofluid containing needle-shaped particles, possibly because the needle-shaped nanoparticles have a high aspect ratio. A higher aspect ratio will correlate with higher surface area to volume ratio, which as previously discussed can lead to a higher thermal conductivity. Zhu et al. [38] measured the thermal conductivity of dimethicone-based nanofluids containing spherical and wire-shaped CuO nanoparticles. There was greater thermal conductivity enhancement in the nanofluid containing wire-shaped particles, as opposed to the nanofluid with sphere-shaped particles. The high aspect ratio of the wire-shaped nanoparticles could contribute to their superior thermal conductivity enhancement, since a higher surface area to volume ratio is the result of the high aspect ratio. Generally, nanoparticles with a high surface area to volume ratio will produce nanofluids with a higher thermal conductivity. The effect of nanoparticle shape requires more research to fully understand the effect.

### 2.5. Effects of Nanoparticle Size on Thermal Conductivity

**Figure 4:**
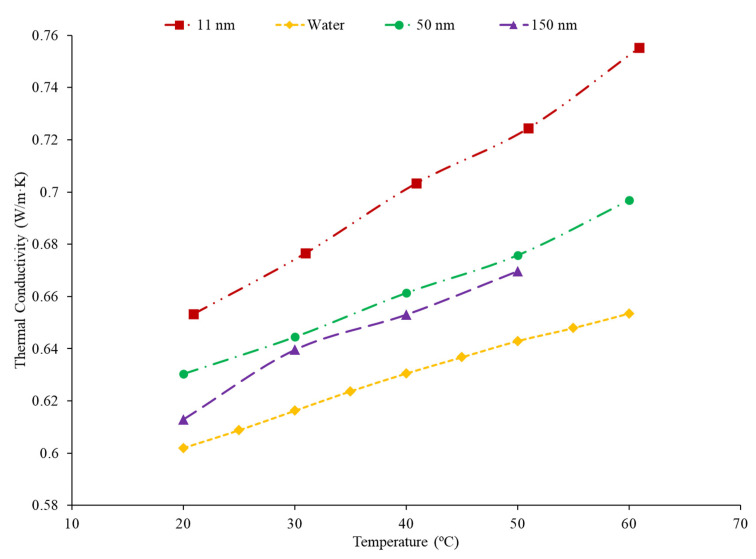
Thermal conductivity of Al_2_O_3_–water nanofluids at a volume fraction of 1% and various sized nanoparticles: 11 nm [22], 150 nm [19], 50 nm [41].

Nanoparticle size can vary greatly and will have an impact on the thermal conductivity of the nanofluid. The majority of research suggests that nanofluids created with smaller nanoparticles will have a higher thermal conductivity, but this is not always the case. Chon et al. [21] studied the thermal conductivity of water-based nanofluids containing 150 nm, 47 nm, and 11 nm Al_2_O_3_ nanoparticles. As the nanoparticle size increased, the thermal conductivity decreased. A decrease in Brownian velocity in the larger nanoparticles could explain the decrease in thermal conductivity as the nanoparticle size increased. The decrease in Brownian velocity will reduce the motion of the particles, and particle motion contributes to greater energy transfer in the nanofluid. Omrani et al. [39] studied the thermal conductivity of water-based nanofluids containing several sizes of multiwalled carbon nanotubes (MWCNT). Each nanofluid experienced an increase in thermal conductivity as the temperature increased. The size of the MWCNTs affected the extent of the increase in thermal conductivity as the greatest increase was in nanofluids containing MWCNTs with a diameter of just above 8 nm and a length of 10–30 μm, which is the nanoparticle with the highest aspect ratio. While the smallest increase in thermal conductivity with temperature was demonstrated in the nanofluid made with nanoparticles with a diameter of just under 50 nm and a length of 0.5–2 μm, these nanoparticles have the smallest aspect ratio. A higher aspect ratio can lead to greater agglomeration, which places the nanoparticles in close contact, allowing for greater heat transfer between the particles. Kwek et al. [40] studied the thermal conductivity of Al_2_O_3_–water nanofluids with a volume concentration of 5% and nanoparticles with diameters of 10 nm, 25 nm, 35 nm, 80 nm, and 150 nm. There was an initial decline in thermal conductivity as the particle diameter began to increase until the diameter reached 35 nm. This decrease can be caused by a decrease in the Brownian motion of the particles as the nanoparticle size increases. Then, the thermal conductivity begins to increase as the nanoparticle size increases beyond 35 nm. This increase in thermal conductivity could be because of an increase in diffusive heat transfer. Diffusive heat transfer allows the nanoparticles to carry heat to other locations throughout the base liquid, depending on many factors including the size and speed of nanoparticles. Figure 4 presents the thermal conductivity as a function of temperature for Al_2_O_3_–water nanofluids with nanoparticle diameters of 11 nm, 50 nm, and 150 nm. The thermal conductivity of the nanofluids decreases as the size of the nanoparticles increases for these nanofluids, for these datasets. This could be caused by a decrease in nanoparticle motion as the size of the nanoparticles increases. In many cases, reducing the nanoparticle size will increase thermal conductivity because the smaller particles have a higher surface area to volume ratio and have more random motion in the base fluid. In some cases, the thermal conductivity will increase with larger particles since the larger particles can hold more energy.

### 2.6. Effects of Surfactant on Thermal Conductivity

**Figure 5:**
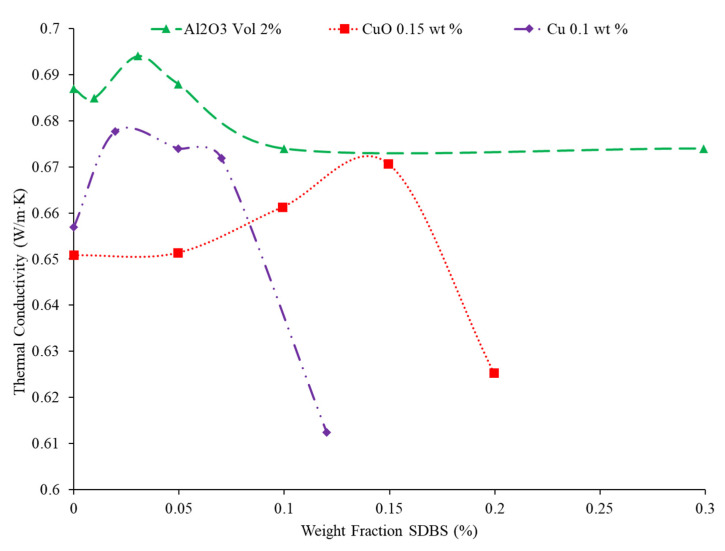
Thermal conductivity of water-based Al_2_O_3_, CuO, and Cu nanofluids with changing concentration of sodium dodecylbenzene sulfonate (SDBS) surfactant: Al_2_O_3_ Vol 2% [41], CuO 0.15 wt % [44], Cu 0.1 wt % [45].

In addition to the base fluid and the nanoparticles, it is common that a nanofluid will contain an additional chemical called a surfactant. The purpose of the surfactant is to stabilize the nanofluid so that it will remain a homogeneous mixture. If a nanofluid becomes unstable, there will be deposition where the nanoparticles separate from the base fluid lead to abrasion and clogging in microchannels. Although surfactants are added to change the stability of the nanofluid, they will also impact the thermal conductivity of the nanofluid. In certain concentrations, the surfactant can increase the thermal conductivity by allowing for greater particle motion; however, in excessively high concentrations, the surfactant can coat the nanoparticles, creating a barrier which prevents heat transfer. Khairul et al. [10] studied the thermal conductivity of water-based Al_2_O_3_ and CuO nanofluids with a changing concentration of surfactant. The volume concentration was varied and sodium dodecyl benzene sulfonate (SDBS) surfactant was used in the measurements to stabilize the nanofluid. The SDBS works by negatively charging the outer surface of the nanoparticles, which then creates an electrostatic force, causing the nanoparticles to repel each other. This repulsive force keeps the nanoparticles separate, creating a more stable nanofluid. The stability of the nanofluids was measured by measuring the zeta potential, which quantifies the surface of the nanoparticles. The higher the surface potential, the more stable the nanofluids. Typically, a zeta potential of greater than +30 mV or less than −30 mV is considered stable. For the nanofluids studied, the optimum zeta potential was found at a surfactant concentration of 0.10 wt% and 0.15 wt% Al_2_O_3_ and CuO, respectively [10]. For both the Al_2_O_3_ and CuO nanofluids, the thermal conductivity increased as the concentration of surfactant increased initially. This could be because the increased stability of the nanofluid allowed for greater particle motion. Eventually, the thermal conductivity reached a maximum for all of the nanofluids, as any additional amounts of surfactant reduced the thermal conductivity. This reduction in thermal conductivity could be caused by surfactant on the particle surface interfering with heat transfer. Das et al. [42] measured the thermal conductivity of TiO_2_–water nanofluids, with cetyl trimethyl ammonium bromide (CTAB) and sodium dodecyl sulfate (SDS) as surfactants. The thermal conductivity of the TiO_2_–CTAB–water nanofluid was lower than the TiO_2_–SDS–water nanofluid. Furthermore, the zeta potential of the two nanofluids was measured to determine the stability of the nanofluids. The SDS and CTAB nanofluids had zeta potentials of −17.8 mV and −21.1 mV, respectively. Therefore, neither nanofluid is totally stable, but the CTAB nanofluids had greater stability. In this case, higher stability did not lead to higher thermal conductivity [42]. Freitas et al. [43] studied the thermal conductivity of multiwalled carbon nanotubes (MWCNT)–water nanofluids and a variety of surfactants added. The surfactants used were Arabic gum (AG) at 0.25 wt%, Triton’s X-100 (TrX) at 0.25 wt%, and MWCNTs with a COOH acid group attached to them. The highest thermal conductivity enhancement at a 1 wt% nanoparticle concentration was the COOH–MWCNT–water nanofluid. The MWCNT–TrX–water nanofluids had the smallest enhancement in thermal conductivity. The Zeta potential of the different nanofluids was tested as well. The COOH–MWCNT nanofluids were the only ones to exhibit a zeta potential in the stable range. These nanofluids also had the highest thermal conductivity, suggesting that the thermal conductivity of the nanofluids can be related to stability in some cases. In general, one can suggest that there is an ideal concentration of surfactant which will yield the highest thermal conductivity. That concentration can be used to optimize nanofluid properties. This effect is demonstrated in Figure 5 where the thermal conductivity of Al_2_O_3_, CuO, and Cu nanofluids is given as a function of concentration of the SDBS surfactant. For all three nanofluids, there is an optimum level of surfactant where the thermal conductivity is maximized. Using different surfactants can create nanofluids with different thermal conductivities for a given nanofluid condition. Additionally, the concentration of surfactant will affect thermal conductivity and there is typically an optimum concentration of surfactant to maximize thermal conductivity.

### 2.7. Thermal Conductivity of Ternary Nanofluids 

**Figure 6:**
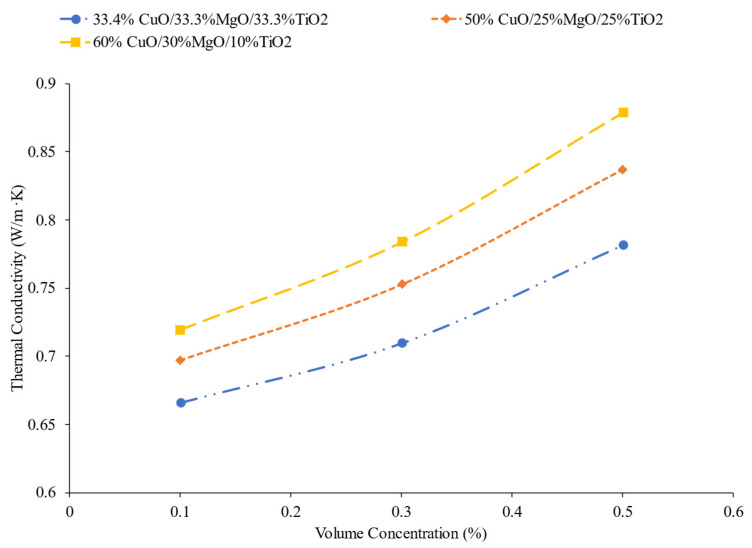
Thermal conductivity of ternary nanofluids as a function of volume concentration with different ratios of nanoparticles: 33.4% CuO/33.3%MgO/33.3%TiO_2_ [49], 50% CuO/25%MgO/25%TiO_2_ [49], 60% CuO/30%MgO/10%TiO_2_ [49].

One of the recent developments in nanofluids has been using more than one kind of nanoparticle. These mixtures of two kinds of nanoparticles are called hybrid nanofluids, while nanofluids with three different nanoparticles are referred to as ternary nanofluids [46]. The primary motivation behind the development of hybrid and ternary nanofluids is that there is a limit to how much the concentration of a nanofluid can be increased before the viscosity becomes too great. Using a combination of particles can further enhance thermal conductivity without creating too high viscosity, optimizing the nanofluid [47]. Utilizing more than one kind of nanoparticle, and the ratios between the different particles, can affect the nanofluid properties. In a heat transfer application, the use of ternary nanofluids has demonstrated an increase in the Nusselt number of nanofluids [48]. Mousavi et al. [49] measured the thermal conductivity of water-based nanofluids with MgO, CuO, and TiO_2_ nanoparticles in different ratios to one another. Figure 6 gives the thermal conductivity of three different nanofluids with a different percent mass of MgO, CuO, and TiO_2_ nanoparticles. For these three nanofluids, the nanofluids with a greater concentration of CuO nanoparticles had a higher thermal conductivity. Of the three nanoparticle materials, CuO had the highest thermal conductivity. That could explain why increasing concentration of CuO increases the thermal conductivity. Additionally, Nabil et al. [50] measure the thermal conductivity of a TiO_2_–SiO_2_ nanofluid in a base fluid mixture of 60% water and 40% ethylene glycol. The thermal conductivity of the nanofluid increased with increases in both the concentration and temperature.

## 3. Review on the Effects of Concentration and Characteristics of Nanofluids on Viscosity

As heat transfer can be a strong function of viscosity sometimes, it is necessary to understand how viscosity is altered when the properties of the nanofluid change. Therefore, a review on this topic is required.

### 3.1. Effects of Base Liquid on Viscosity

**Figure 7:**
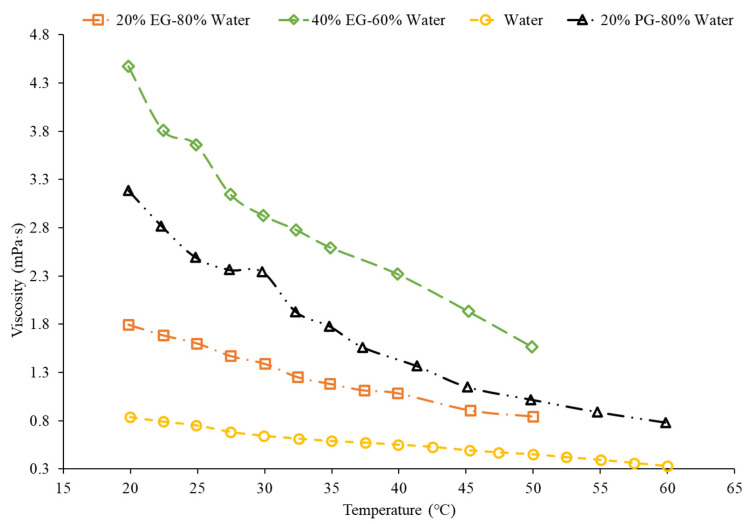
Viscosity as a function of temperature for various base liquids: Water [33], 20% EG–80% Water [54], 40% EG–60% Water: Water [33], 20% EG–80% Water [54], 40% EG–60% Water [54], 20% PG–80% Water [55].

Wang et al. [51] studied the viscosity of MWCNT–([HMIM]BF4) and graphene–([HMIM]BF) nanofluids with temperatures ranging from 25 °C to 75 °C. As the temperature rose, the viscosity decreased. The viscosity of the graphene nanofluid de-creased from 217.4 mPa*s down to 40.6 mPa*s with a rising temperature. The pure ([HMIM]BF4) had a higher viscosity than the ionic liquid-based ([HMIM]BF4) nanofluid. The reason for this is due to the self-lubrication of the MWCNTs and graphene in the base fluid. The pure ([HMIM]BF4) had a higher viscosity than the ionic liquid-based ([HMIM]BF4) nanofluid. The reason for this is due to the self-lubrication of the MWCNTs and graphene in the base fluid. Al-Waeli et al. [52] studied viscosity measurements on SiC–35% ethylene glycol–65% water, SiC–water, and SiC–35% propylene glycol–65% water nanofluids. The viscosity changed with the base fluid used. The SiC–35% ethylene glycol–65% water, SiC–water, and SiC–35% propylene glycol–65% water showed 12.66%, 0.063%, and 16.66% increases in viscosity, respectively, when compared to pure water. Ethylene glycol and propylene glycol had higher viscosities than pure water, so the nanofluids that had a mixture of propylene glycol and ethylene glycol with water had a large increase in viscosity compared to pure water. Kumar et al. [53] measured the viscosity of hybrid nanofluids made with CuO and Al_2_O_3_ nanoparticles with water–ethylene glycol mixture and water–propylene glycol mixture. The viscosity of the nanofluid increased as the concentration of propylene glycol and ethylene glycol increased. This is due to propylene glycol and ethylene glycol having a higher viscosity than water. Moreover, it was studied that the water–propylene glycol mixture has a higher viscosity than that of the water–ethylene glycol mixture. This is due to the fact that propylene glycol as a base liquid has a higher viscosity than that of ethylene glycol. Figure 7 shows that the Fe_3_O_4_ nanofluid with 20% PG–80% water has a higher viscosity as a function of temperature than the 20% EG–80% water, supporting the idea that PG has a higher viscosity as a base liquid than EG.

### 3.2. Effects of Nanoparticle Material on Viscosity

**Figure 8:**
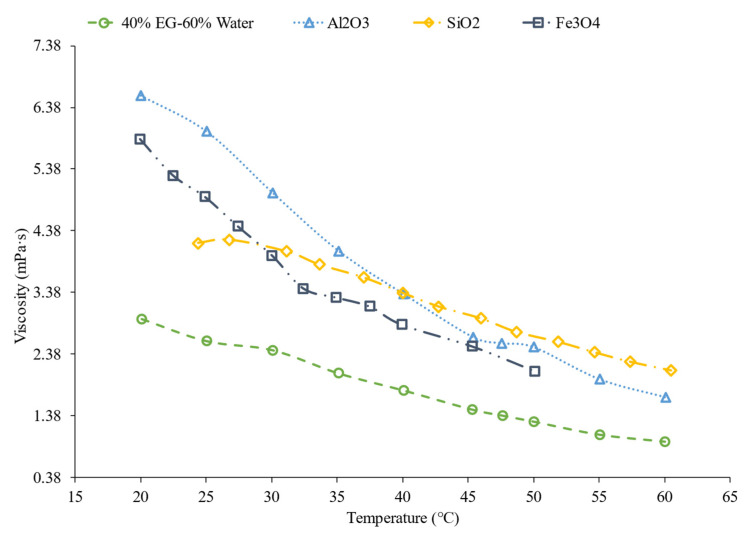
Viscosity as a function of temperature for various materials consisting of Al_2_O_3_, SiO_2_, and Fe_3_O_4_: Al_2_O_3_ [21], Fe_3_O_4_ [54], SiO_2_ [57].

Additionally, the choice of material within nanofluids affects the viscosity of nanofluids. Yiamsawas et al. [56] studied the viscosity of TiO_2_ and Al_2_O_3_ water-based nanofluids. A capillary tube viscometer was used to measure the changes in viscosity. The nanofluids studied had concentrations ranging from 1% to 4%, with temperatures ranging from 15 °C to 60 °C. Across the range of temperatures, the nanofluids decreased in viscosity and the TiO_2_ nanofluid had viscosity that was lower than the Al_2_O_3_ nanofluid. For the nanofluids with a 1% volume concentration, the TiO_2_ nanofluids had an average viscosity that was 19.2% lower than the Al_2_O_3_ nanofluid across the temperature range studied. Nguyen et al. [51] studied the viscosity of water-based Al_2_O_3_ and CuO nanofluids at concentrations of 1%, 4%, 7%, and around 9%. The Al_2_O_3_–water had a lower viscosity than the CuO–water nanofluids. The Al_2_O_3_ nanofluid at a temperature of 30 °C with volume concentrations of 1%, 4%, 7%, and 9.4% had a viscosity of 0.8 mPa·s, 1.3 mPa·s, 1.7 mPa·s, and 3.6 mPa·s, respectively. The CuO nanofluid at a temperature of 30 °C with volume concentrations of 1%, 4.5%, 7%, and 9% had a viscosity of 0.9 mPa·s, 1.5 mPa·s, 3.1 mPa·s, and 6.5 mPa·s, respectively. Sundar et al. [15] studied the viscosity of ND–water nanofluids with volume concentrations of 0.2%, 0.4%, 0.6%, 0.8%, and 1% with temperatures ranging from 20 °C to 60 °C. The important takeaway to note is that the viscosity decreased as the temperature increased, as with all other material examined in the given references. In Figure 8 below, it can be concluded that the viscosity of the particular material depends on what temperature range is being studied. For example, Al_2_O_3_ has a higher viscosity than SiO_2_ under 40 °C, but above 40 °C, it is the other way around.

### 3.3. Effects of Nanoparticle Concentration on Viscosity

**Figure 9:**
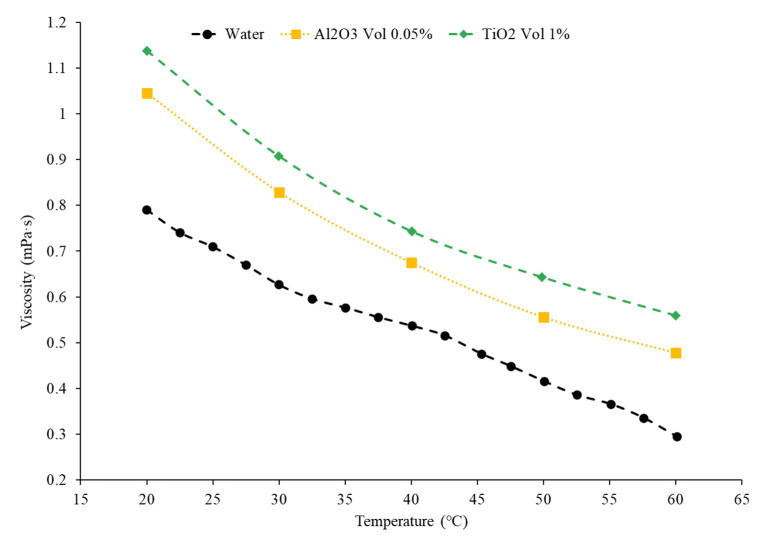
Viscosity as a function of temperature for concentrations of 0.05% and 1%: Al_2_O_3_ Vol 0.05% [24], TiO_2_ [56].

Furthermore, studies have concluded that there is an increase in the viscosity of a nanofluid when the concentration of nanoparticles increases. Sundar et al. [33] studied the viscosity of Fe_3_O_4_–water nanofluids with varying particle concentrations of 0.2% to 2%. There was an increase in the viscosity of the nanofluid as the volume concentration increased. The increase in viscosity with volume concentration increasing could be due to an increase in the interaction between particles. Gao et al. [58] studied the viscosity of Fe_3_O_4_–water nanofluids at volume concentrations ranging from 0.05% to 2%. The temperature ranged from 10 °C to 65 °C. The viscosity of the nanofluids increased with increasing volume concentration. Nguyen et al. [59] looked into the effect of nanoparticle volume fraction on the viscosities of Al_2_O_3_–water and CuO–water nanofluids. Particle diameters for these nanoparticles consisted of 29 nm for the CuO–water and 36 nm/47 nm for the Al_2_O_3_–water nanofluids. They were placed in a room temperature of 25 °C. The volume fractions ranged from 0.15% to 13%. The nanofluid viscosity increased with nanoparticle volume fraction increasing. For example, the relative viscosity of the 47 nm water–Al_2_O_3_ nanofluid rose with volume concentration. The relative viscosity values were 1.12, 1.6, 3.0, and then 5.2 for particle volume concentrations of 1%, 4%, 9%, and 12% respectively. Malekzadeh et al. [60] studied the viscosity of Fe_3_O_4_–water nanofluids. Volume concentrations of 0.1% to 1% were studied with temperatures ranging from 25 °C to 45 °C. The increase in viscosity from the rise in volume concentration can be explained by the higher levels of molecular interaction between the nanoparticles and base liquid when higher concentrations were used. Godson et al. [34] studied the viscosity of Ag–water nanofluids with varying volume fractions of 0.3%, 0.6%, and 0.9% and the viscosity increased with volume concentration increasing. Figure 9 below shows the effect of nanoparticle concentration on viscosity. As can be seen by the graph, as the concentration of nanoparticles increases, the viscosity increases as well.

### 3.4. Effects of Nanoparticle Shape on Viscosity

Additionally, the shape of nanoparticles does not affect the viscosity of the nanofluid greatly. Main et al. [37] studied the viscosity of rod, needle, and sphere-shaped Al_2_O_3_-1-Butyl-3-methylimidazolium bis(trifluoromethylsulfonyl) imide ([C4mim] [NTf2]) nanofluids. The viscosity did not vary greatly between the different shaped nanoparticles. Furthermore, Zhu et al. [38] studied the viscosity of the CuO–dimethicone nanofluid with wire and spherical-shaped nanoparticles. At a steady temperature of 25 °C, the viscosity of the nanoparticles increased with volume concentration. However, there was no clear change in the viscosity of the two nanofluids based on the different shaped nanoparticles used.

### 3.5. Effects of Nanoparticle Size on Viscosity

**Figure 10:**
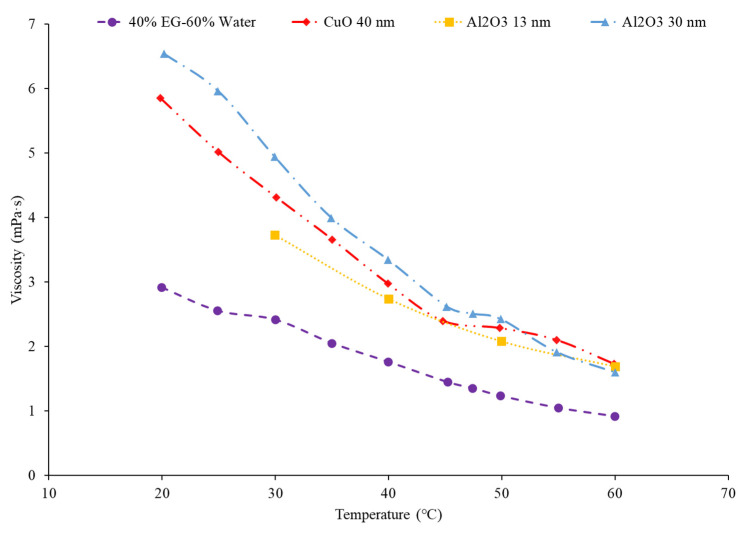
Viscosity as a function of temperature with various sized nanoparticles: Al_2_O_3_ 30 nm [21], CuO 40 nm [63], Al_2_O_3_ [64].

Furthermore, the effect of nanoparticle size on the nanofluid viscosity has been studied. Researchers found that as the size of a nanoparticle increases, the viscosity decreases. Kwek et al. [40] studied the viscosity of Al_2_O_3_–water nanofluids with a volume concentration of 5% and nanoparticle diameters of 10 nm, 25 nm, 35 nm, 80 nm, and 150 nm. The viscosity decreased as the nanoparticle size increased, until the nanoparticle size reached 85 nm. Then, the viscosity just approached a constant value. The viscosity decreases as the nanoparticle size increases due to the fact that smaller nanoparticles like to group together and create more clusters when compared to larger nanoparticles. Particle agglomeration is the idea of nanoparticles forming into clusters within a fluid, which in turn leads to higher viscosities for these types of fluids. Jia-Fei et al. [61] also examined how the viscosity of a nanofluid is affected by nanoparticle size. SiO_2_–water nanofluids were used and the nanofluids had volume concentrations of 0.1%, 0.2%, 0.4%, 0.8%, 1.2%, 1.6%, and 2%. They had nanoparticle diameters of 7 nm, 12 nm, 16 nm, 20 nm, and 40 nm. At each concentration tested, as the nanoparticle size increased, the viscosity of the nanofluid decreased. Rudyak et al. [62] studied the viscosity of Al_2_O_3_–water, TiO_2_–water, and SiO_2_–water nanofluids at 2% volume fraction. The nanoparticles ranged from 10 nm to 150 nm. All the nanofluids tested showed that as the nanoparticle size increased, the viscosity decreased. In Figure 10, it can be seen that as the nanoparticle size tends to increase for the Al_2_O_3_ nanofluid, the viscosity increases as well. However, as the nanoparticle size increases at a temperature above around 57 °C, the viscosity actually decreases. Therefore, the effects of nanoparticle size on the viscosity of a nanofluid cannot have a definite trend as it essentially depends on the temperature range being studied.

### 3.6. Effects of Surfactant on Viscosity

**Figure 11:**
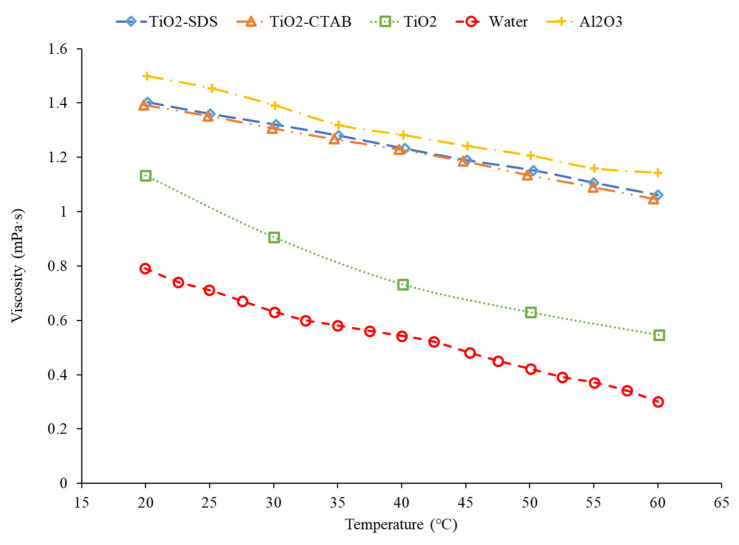
Viscosity as a function of temperature with varying surfactants: Al_2_O_3_ [41], TiO_2_–CTAB [42], TiO_1_-SDS [42], TiO_2_ [56].

In addition, research has proven how the addition of a surfactant plays a role on the viscous properties of a nanofluid. Khairul et al. [10] studied the viscosity of Al_2_O_3_–water and CuO–water nanofluids with SDBS surfactant. The viscosities of the CuO–water and Al_2_O_3_–water nanofluids decreased as the concentration of the surfactant increased, but there was somewhat of a fluctuation, resulting in an indefinite conclusion on the exact relationship. The Al_2_O_3_–water nanofluid had a minimum viscosity at 0.1 wt% of SDBS and the CuO nanofluid had a minimum viscosity at 0.15 wt% of SDBS. Das et al. [41] also measured the viscosity of TiO_2_–water nanofluids using CTAB and SDS as surfactants. A range of temperatures, from 20 °C to 60 °C, was used. The nanofluids had volume concentrations of 0.1%, 0.5%, and 1%. The viscosity for the TiO_2_–CTAB–water nanofluid was fairly close to the TiO_2_–SDS–water nanofluid. Figure 11 shows that the viscosity increases with the use of a surfactant as the TiO_2_–SDS and TiO_2_–CTAB have a higher viscosity than just the TiO_2_ nanofluid itself.

### 3.7. Viscosity of Ternary Nanofluids

**Figure 12:**
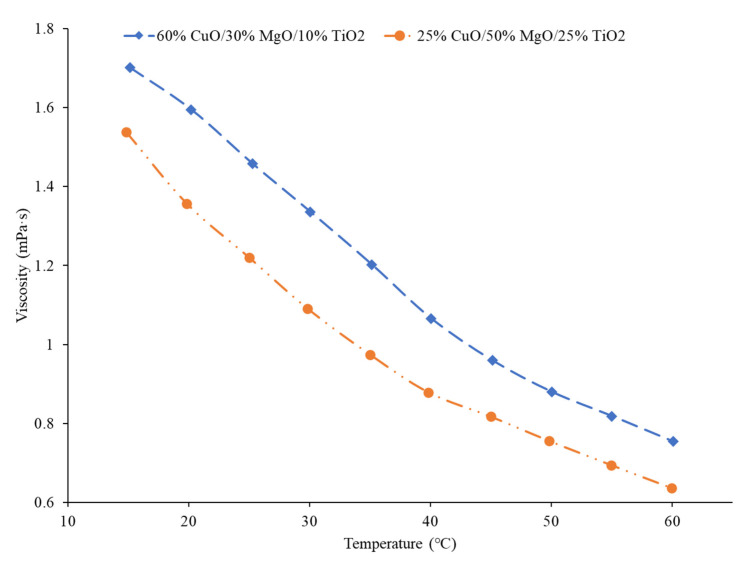
Viscosity of ternary nanofluids as a function of temperature with different ratios of nanoparticles: 60% CuO/30% MgO/10% TiO_2_ [49], 25% CuO/50% MgO/25% TiO_2_ [49].

The viscosity of hybrid and ternary nanofluids has been a major area of study as well as thermal conductivity. Ahmed et al. [65] measured the viscosity of a water-based ternary nanofluid with equal parts of ZnO, TiO_2_, and Al_2_O_3_ nanoparticles. As with other water-based nanofluids, as the concentration increased, the viscosity increased as well. An increase in temperature, however, led to a reduction in viscosity. Mousavi et al. [49] measured the viscosity of water-based nanofluids with MgO, CuO, and TiO_2_ nanoparticles in different ratios to one another. Figure 12 gives the viscosity of two different nanofluids with a different percent mass of MgO, CuO, and TiO_2_ nanoparticles. The nanofluid with the highest percentage of CuO had the highest viscosity in this case. In Figure 6, it was shown that the nanofluid with the most CuO also had the highest thermal conductivity. Therefore, there may be a trade-off from the high thermal conductivity. Sahoo and Kumar [66] measured the viscosity of water-based nanofluids with Al_2_O_3_, CuO, Al_2_O_3_–CuO hybrid; Al_2_O_3_–TiO_2_ hybrid; and Al_2_O_3_, CuO, TiO_2_ ternary nanofluids. The nanofluid with the highest viscosity was the CuO nanofluid. The ternary nanofluid had the second highest viscosity and had a higher viscosity than either of the hybrid nanofluids. In several cases, CuO produced high-viscosity nanofluids. Additionally, in some cases, a change in the number of nanoparticle materials can increase the viscosity.

## 4. Review on the Optimization of Effects of Nanoparticles

**Figure 13:**
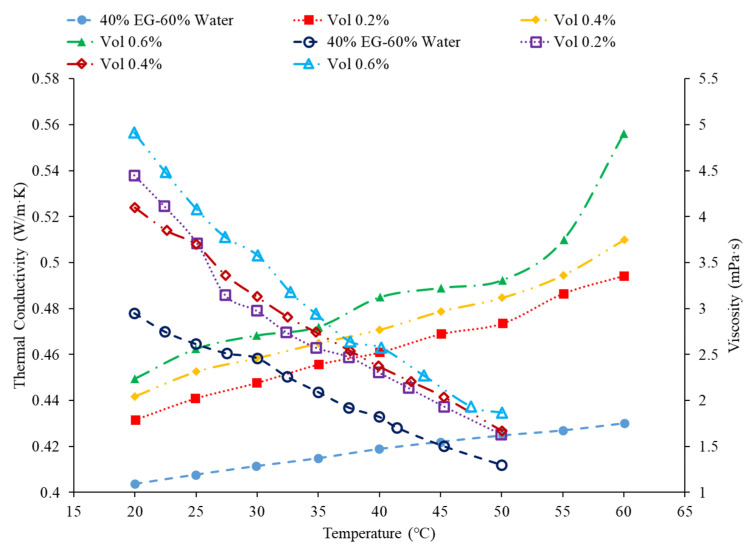
Thermal conductivity and viscosity of Fe_3_O_4_–40% ethylene and glycol–60% water nanofluids at several concentrations at several volume concentrations: Vol 0.2% [67], Vol 0.2% [67], Vol 0.4% [67], Vol 0.4% [67], Vol 0.6% [67], Vol 0.6% [67].

In order to achieve the ideal thermal properties of a nanofluid, the thermal conductivity should be maximized, while the viscosity is minimized [1]. One method of achieving this is increasing the temperature of the nanofluid since the temperature increases as the viscosity decreases, while the thermal conductivity increases. This effect is demonstrated in Figure 13, where the thermal conductivity and viscosity for Fe_3_O_4_–40% ethylene and glycol–60% water nanofluids are given as functions of temperature for several volume concentrations. As the temperature is increased, the thermal conductivity increases while the viscosity decreases [67]. Additionally, both the thermal conductivity and viscosity increase as the nanofluid concentration increases. The increase in thermal conductivity is beneficial to the thermal performance, while the increase in viscosity decreases the heat transfer ability of the fluid by suppressing random motion of molecules and particles. The random motion of molecules and particles in a nanofluid is thought to be a primary mechanism of energy transfer [68]. Therefore, the concentration of the nanofluid must be optimized based on whether the increase in thermal conductivity or viscosity is more dominant. Figure 13 also demonstrates an additional benefit of increased nanofluid temperature. The difference in viscosity between nanofluids with different concentrations is significantly diminished at higher temperatures. Additional parameters, such as the use of a surfactant and the nanoparticle size, can be considered to optimize a nanofluid to achieve the more effective physical properties [69]. It is possible to simultaneously increase the thermal conductivity and decrease the viscosity by increasing the temperature of the nanofluid [30]. Increasing the nanofluid concentration will generally increase both thermal conductivity and viscosity; therefore, it is necessary to balance the positive effect on thermal conductivity, while considering the potential negative effect of an increased viscosity [31]. 

## 5. Review on the Effects of Concentration and Characteristics of Nanofluids on the Heat Transfer Coefficient

With the indirect effects of nanofluid properties through thermal conductivity and viscosity on the heat transfer discussed, the effects on the heat transfer coefficient can now be considered.

### 5.1. Effects of Base Liquid on the Heat Transfer Coefficient

**Figure 14:**
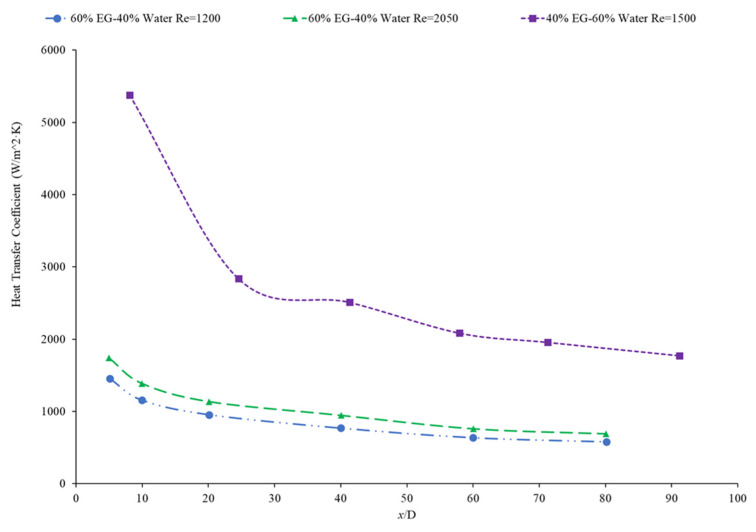
Heat transfer coefficient as a function of nondimensionalized distance for TiO_2_ nanofluids at 0.4% volume fraction and base liquids of 60% ethylene glycol (EG)-40% water and 40% ethylene glycol (EG)-60% water: 60% EG-40% Water Re = 1200 [77], 60% EG-40% Water Re = 2050 [77], 40% EG-60% Water Re = 1500 [78].

Bayat and Nikseresht [70] studied the effect of pure water, pure ethylene glycol, and water–ethylene glycol mixture on the heat transfer coefficient of Al_2_O_3_ nanoparticles. The relative heat transfer coefficient was higher with ethylene glycol as a base liquid compared to water. In addition, when looking at the ethylene glycol–water mixture nanofluid, the relative heat transfer coefficient, compared to the base fluid, increased when increasing the mass of ethylene glycol. Maiga et al. [71] studied two different nanofluid bases, both containing Al_2_O_3_ nanoparticles. The ethylene glycol-based nanofluid was shown to produce a higher heat transfer coefficient than the water-based nanofluid at similar concentrations and Reynolds numbers. The water-based nanofluid with a Reynolds number of Re = 500 saw heat transfer coefficients of 400 to 700 (units), whereas for the ethylene glycol-based fluid with Re = 631, the heat transfer coefficient ranged from 2000 to 6000 (units). Jyothirmayee Aravind and Ramaprahu et al. [72] measured the heat transfer coefficient of graphene-wrapped MWCNT nanofluids with base fluids of water and ethylene glycol. The nanofluids measured were at a volume fraction of 0.01% and 0.02%. At a Reynolds number of 10,000 and a volume fraction 0.02%, the water-based nanofluid had an average heat transfer coefficient of 2500 $W/\left(m^{2}\cdot K\right)$, while the ethylene glycol-based nanofluid had an average heat transfer coefficient of 626 $W/\left(m^{2}\cdot K\right)$. The heat transfer coefficient of pure water is also higher than the heat transfer coefficient of pure ethylene glycol, suggesting that the heat transfer coefficient of the nanofluid is largely dependent on the heat transfer coefficient of the base fluid. Ebrahimnia-Bajestan et al. [73] studied water-based and 60% ethylene glycol–40% water mixture-based nanofluids as coolants for solar heat exchangers. Their research also led to the conclusion that the thermophysical properties of the base fluid greatly affects the heat transfer characteristics of their nanofluids. At a nanoparticle concentration of 2.3%, they found water-based nanofluids to have a higher heat transfer coefficient when the Reynolds number was below 1000. Past that point, however, the ethylene glycol and water-based nanofluid had higher heat transfer coefficient readings. Further study found that, at a Reynolds number above 1000, the ethylene glycol and water mixture had a higher heat transfer coefficient as long as the particle concentration was less than 2%. The high viscosity of the ethylene glycol–water mixture causes the ethylene glycol–water mixture-based nanofluids to have a low heat transfer coefficient at a high concentration and a low Reynolds number where the effect of viscosity is most significant. Baby and Ramaprabhu [74] measured the heat transfer coefficient of water and ethylene glycol-based silver-decorated functionalized hydrogen-induced exfoliated graphene (Ag/HEG) nanofluids. The experiments were conducted in a stainless-steel tube with nanofluids at volume fractions of 0.005% and 0.01%. The Reynolds numbers studied were 4500, 8700, and 15,500 for the water-based nanofluids and 250, 500, and 1000 for the ethylene glycol-based nanofluids. The enhancement in heat transfer coefficient as compared to the base fluid was greater in the ethylene glycol-based nanofluids than the water-based nanofluids. Therefore, the effect of adding nanoparticles is greater in ethylene glycol-based nanofluids. Aravind et al. [75] measured the heat transfer coefficient of functionalized multi-wall carbon nanotube (f-MWCNT) nanofluids with base fluids of water and ethylene glycol. The nanofluids were tested at volume fractions of 0.03% and 0.005%. The water-based nanofluids had a higher heat transfer coefficient than the ethylene glycol-based nanofluids. The water-based nanofluids also had a higher thermal conductivity and a lower viscosity than the ethylene glycol-based nanofluids. The superior thermophysical properties of the water-based nanofluids for heat transfer would explain the higher heat transfer coefficient. Sundararaj et al. [76] measured the heat transfer coefficient of Al_2_O_3_–kerosene nanofluids. The experiments were conducted in a copper tube 4 mm in diameter and 1 m in length using nanofluids with a volume fraction of 0.01% and 0.05%. In the kerosene-based nanofluids, as both the concentration and the Reynolds number increased, the heat transfer coefficient increased. Figure 14 shows the heat transfer coefficient as a function of nondimensionalized distance for TiO_2_ nanofluids with base fluids of 60% ethylene glycol–40% water and 40% ethylene glycol–60% water at volume fractions of 0.4%. The nanofluid with a higher concentration of ethylene glycol had a lower heat transfer coefficient. This agrees with the idea that water–based nanofluids have a higher heat transfer coefficient than ethylene glycol-based nanofluids. As shown in Figure 1, water-based nanofluids also had a higher thermal conductivity than ethylene glycol-based nanofluids. Since the heat transfer coefficient is dependent on the fluid’s thermal conductivity, the higher thermal conductivity of water compared to ethylene glycol will cause water-based nanofluids to have a higher heat transfer coefficient. Therefore, in a water–ethylene glycol mixture, a higher concentration of water will increase the heat transfer coefficient. Water and ethylene glycol are the two primary base fluids used in nanofluid heat transfer studies. In many cases, water-based nanofluids will have a higher heat transfer coefficient due to the higher thermal conductivity and lower viscosity of water. The relative heat transfer coefficient compared to the base fluid is often higher in ethylene glycol-based nanofluids, indicating there is a great effect from adding nanoparticles to ethylene glycol.

### 5.2. Effects of Nanoparticle Material on the Heat Transfer Coefficient

**Figure 15:**
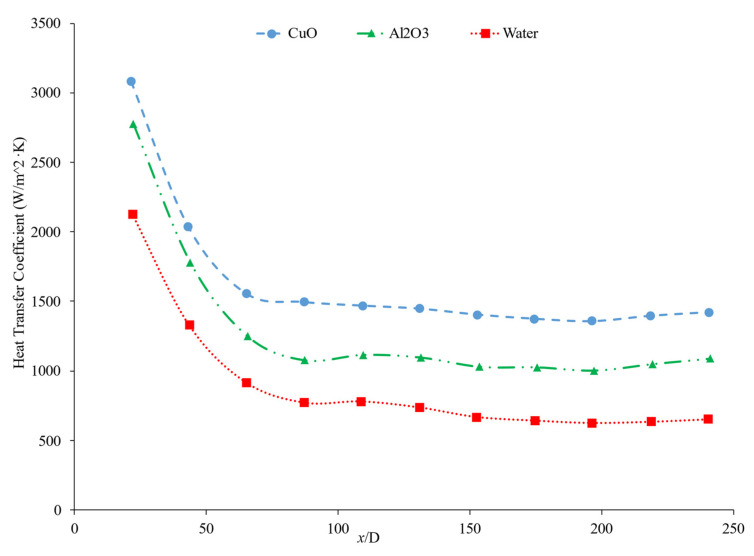
Heat transfer coefficient as a function of nondimensionalized distance for water-based CuO and Al_2_O_3_ nanofluids at 0.5 wt% and Re = 1400: CuO [85], Al_2_O_3_ [85].

The thermal boundary layer of the nanofluid is smallest when the heat transfer coefficient is the largest. Mohan et al. [79] found, while running different nanofluids through a thermally insulated pipe, that an alumina nanofluid had an average thermal boundary thickness of 0.43 mm, while a similar concentration of a silica nanofluid had an average boundary thickness of 0.67 mm. The base fluid was DI water and, by itself, had an average thermal boundary layer thickness of 0.56 mm. The heat transfer coefficient was enhanced by 15.8% on average when using the alumina nanofluid at a volume fraction of 0.02% as opposed to the base fluid, while it decreased by 12.4% when the silica nanofluid was used at the same concentration. Titania nanofluids were found to have lower heat transfer coefficients compared to alumina nanofluids in a study by Utomo et al. [80]. In an experiment performed by Ding et al. [81] several different water-based nanofluids were run through a 3.97 mm pipe fitted with thermocouples. In this experiment, it was observed that, given equal concentrations of nanoparticles at 0.1 wt% and 30◦C, water–carbon-based nanofluids saw the greatest enhancement of thermal conductivity and was followed in decreasing order by aqueous titanate, aqueous titania, and, lastlym by ethylene glycol-based titania nanofluids and aqueous-based nano-diamond nanofluids, which were found to give little enhancement, if any, under these conditions. Ebrahimnia-Bajestan et al. [73] saw very predictable results among water-based nanofluids with three separate nanoparticle types. The particle types with higher thermal conductivity saw higher heat transfer coefficient values and saw greater enhancement as the Reynolds number increased. Heris et al. [82] had similar results in his oil-based nanofluids. Higher thermal conductivity particles resulted in higher heat transfer enhancement. Vajjha et al. [83] measured the heat transfer coefficient of CuO–water and Al_2_O_3_–water nanofluids in the laminar flow regime in a flattened tube automobile radiator with nanofluids that have a concentration up to 6%. There was a negligible difference in the average heat transfer coefficient of CuO and Al_2_O_3_ nanofluids. Pattanayak et al. [84] measured the heat transfer coefficient of water-based nanofluids containing CuO, TiO_2_, Al_2_O_3_, and ZnO nanoparticles in a double pipe heat exchanger. The concentration of the nanofluids also varied from 0.025% to 0.1%. At a volume fraction of 0.1%, the greatest increase in heat transfer coefficient was measured for the TiO_2_ nanofluids followed by the CuO, ZnO, and Al_2_O_3_ nanofluids. Khairul et al. [85] measured the heat transfer coefficient of water-based Al_2_O_3_ and CuO nanofluids. The experiments were performed in a 1-m-long stainless-steel tube with an inner diameter of 4.57 mm. In Figure 15, the measurements were given for heat transfer coefficient as a function of nondimensionalized distance for both the CuO–water and Al_2_O_3_–water nanofluids at 0.5 wt% with a Reynolds number of 1400. The CuO nanofluid had a higher heat transfer coefficient than the Al_2_O_3_ nanofluid. CuO nanoparticles have a higher thermal conductivity than Al_2_O_3_ nanoparticles, which leads to the CuO nanofluid having a higher thermal conductivity as well. Both nanofluids demonstrate a significant decrease in heat transfer coefficient as the axial distance increases. This is due to the increase in thickness of the thermal boundary layer as the axial distance increases. Brownian motion of nanoparticles can affect the thermal boundary layer which will contribute to the increased heat transfer coefficient of the nanofluids compared to the base fluid as well.

**Figure 16:**
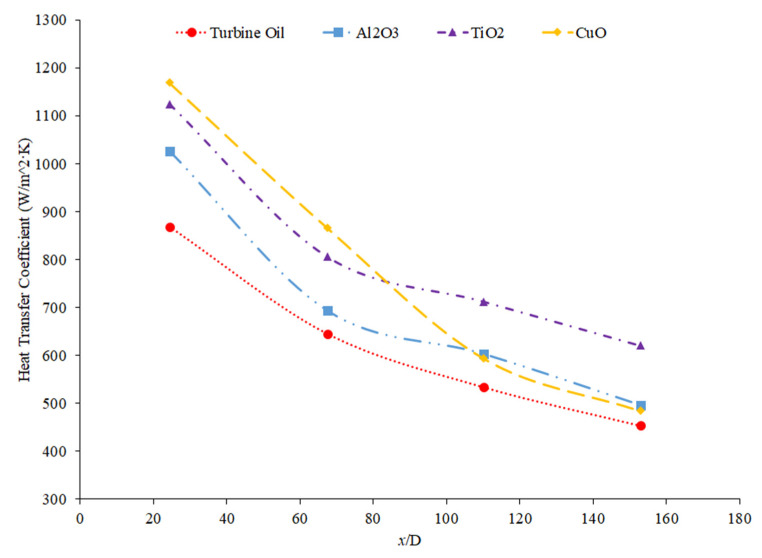
Heat transfer coefficient as a function of location for turbine oil-based Al_2_O_3_, CuO, and TiO_2_ nanofluids at a Reynolds number of 750 and volume fraction of 0.5%: Turbine Oil [82], Al_2_O_3_ [82], TiO_2_ [82], CuO [82].

Hussien et al. [86] measured the heat transfer coefficient of water-based nanofluids containing multi-walled carbon nanotubes (MWCNTs) and hybrid nanofluids with MWCNTs and graphene nanoplatelets (GNPs). The experiments were conducted in a 1.1-mm-diameter brass tube with a length of 270 mm, and the flows had Reynolds numbers between 200 and 470. At a weight percent of 0.125, the MWCNT–GNP hybrid nanofluid had a higher heat transfer coefficient than the MWCNT nanofluid. Both nanofluids had an increase in the heat transfer coefficient compared to pure water. In both nanofluids, as the concentration of the nanofluid increased, the heat transfer coefficient increased as well. The increase in the heat transfer coefficient of the nanofluids is due to the enhanced thermophysical properties of the nanofluids compared to the base fluid and the effects of Brownian motion. Heris et al. [82] measured the heat transfer coefficient of turbine oil-based nanofluids with CuO, TiO_2_, and Al_2_O_3_ nanoparticles. The experiment was conducted in a 1.3 m copper tube with an inner diameter of 8 mm. Figure 16 gives the heat transfer coefficient as a function of location for the turbine oil-based CuO, TiO_2_, and Al_2_O_3_ nanofluids at a Reynolds number of 750 and all nanofluids at a volume fraction of 0.5%. The heat transfer coefficient of the three nanofluids is significantly influenced by the location. All three nanofluids have a heat transfer coefficient much higher than the base fluid in the entrance region due to the random motion of nanoparticles preventing the formation of a thermal boundary layer. Initially, when looking at Figure 16, the CuO nanofluids had the highest heat transfer coefficient, which could possibly be attributed to the fact that CuO nanoparticles have a higher thermal conductivity than Al_2_O_3_ and TiO_2_, contributing to greater heat transfer. Further down the tube, the TiO_2_ nanofluid had the highest heat transfer coefficient. Of the three nanoparticles, the TiO_2_ nanoparticles were the smallest, which could contribute to greater random motion, further disrupting the development of the thermal boundary layer more than the other materials, leading to a higher heat transfer coefficient than the other two nanoparticles. Therefore, despite the lower thermal conductivity of the TiO_2_ nanoparticles, the high particle motion that comes from smaller nanoparticles gives a high heat transfer coefficient by disrupting the thermal boundary layer. Overall, the size and material can both affect the nanofluid properties simultaneously. There is not necessarily a nanoparticle material that produces the highest heat transfer coefficient nanofluids, but in many cases, TiO_2_ and CuO nanoparticles create high heat transfer coefficient nanofluids.

### 5.3. Effects of Nanoparticle Concentration on the Heat Transfer Coefficient

**Figure 17:**
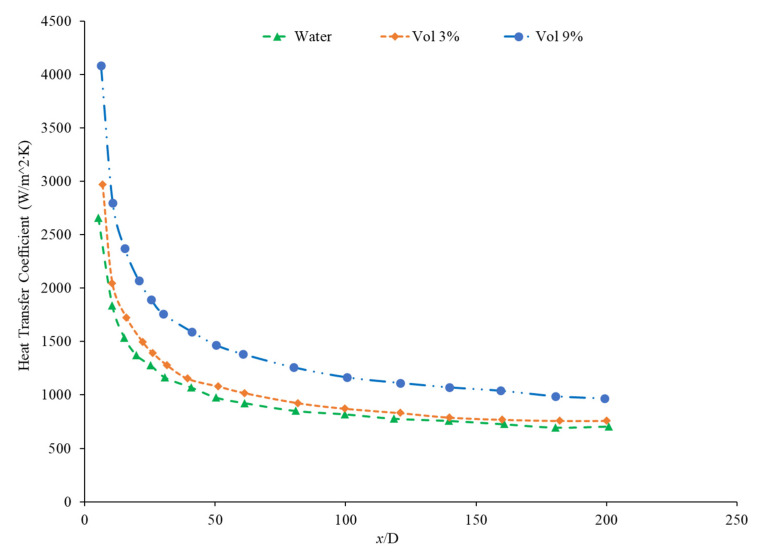
Heat transfer coefficient as a function of axial distance (x/D) for volume fractions of 0%, 3%, and 9% for Al_2_O_3_–water-based nanofluids: Water 0% [70], Vol 3% [70], Vol 9% [70].

Nanoparticle concentration is also important due to its effects on the heat transfer coefficient of nanofluids. Both thermal conductivity and viscosity are impacted by concentration, making it vital to understanding the thermal transport characteristics of the nanofluid. Zamzamian et al. [87] studied the effect of increasing the concentration of nanoparticles in Al_2_O_3_–ethylene glycol and CuO–ethylene glycol nanofluids on the convective heat transfer coefficient. The nanofluids were studied in turbulent flow in a double pipe and plate heat exchangers. As the nanoparticle concentration increased, the convective heat transfer coefficient increased. This increase in heat transfer coefficient was caused by an increase in thermal conductivity and chaotic motion of the particles in the fluid. Additionally, Esmaeilzadeh et al. [88] investigated the effect of increasing the volume concentration of 15 nm Al_2_O_3_ nanoparticles in a base liquid of water on the convective heat transfer coefficient. The convective heat transfer coefficient was enhanced by 6.8% and 19.1% compared to pure water with a nanoparticle volume concentration change of 0.5% to 1%, respectively. In addition, Bhanvase et al. [78] studied the effect of increasing the volume fraction of TiO_2_ nanoparticles on the convective heat transfer coefficient. Nanofluids were prepared with TiO_2_ nanoparticles with a particle size below 100 nm and volume fractions less than 0.5%. A base liquid mixture of 40% ethylene glycol (EG) and 60% water was used. There was an increase in the convective heat transfer coefficient and an increase in the volume fraction of the nanoparticles used. The nanoparticle can help facilitate the transition of the flow from laminar to turbulent, increasing the heat transfer coefficient by increasing random motion in the fluid. Additionally, Bayat and Nikseresht [70] also studied the effect of volume concentration on the heat transfer coefficient. Volume concentrations for Al_2_O_3_–water-based nanofluid ranged from 0 to 9%. Figure 17 below shows the behavior of the heat transfer coefficient as a function of axial distance (x/D) with varying volume fractions of Al_2_O_3_–water-based nanofluid from measurements [70].

**Figure 18:**
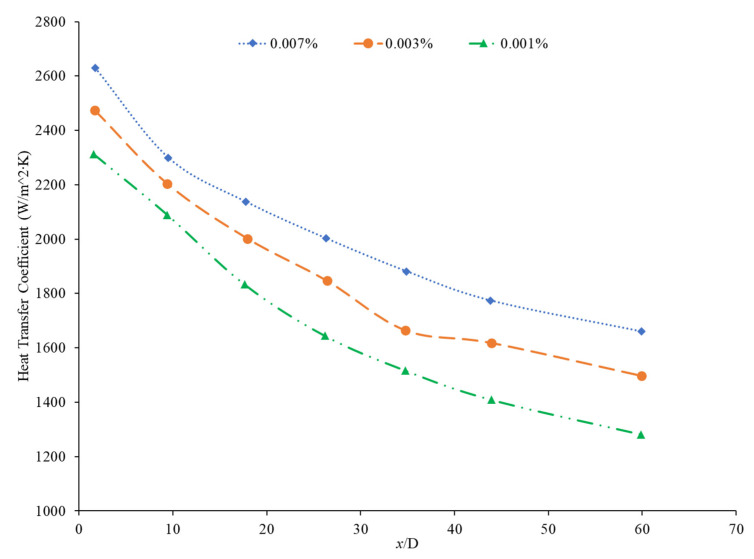
Convective heat transfer coefficient as a function of axial distance (x/D) for SiO_2_–water-based nanofluids varying in volume concentration: SiO_2_ 0.007% [90], SiO_2_ 0.003% [90], SiO_2_ 0.001% [90].

In the study performed by Mohan et al. [79], the concentration of an SiO_2_–water nanofluid was increased from 0.01% to 0.02% by volume. The lower concentration was found to have a higher heat transfer coefficient. In this same study, an Al_2_O_3_–water nanofluid had its heat transfer coefficient increase as its concentration increased. There are two possible explanations for this behavior. Since Al_2_O_3_ is a superior thermal conductor to SiO_2_, an increased concentration of Al_2_O_3_ would more effectively increase the thermal conductivity of the water. Additionally, the Al_2_O_3_–water nanofluid had a smaller thermal boundary layer than the SiO_2_–water nanofluid, facilitating greater heat transfer. Another study by Hwang et al. [89] found increasing concentration of Al_2_O_3_ nanoparticles in water which resulted in an 8% increase in the heat transfer coefficient as the concentration was raised from 0.01% to 0.3%. Akhavan-Zanjani et al. [16] also found that increased concentration of graphene nanoparticles in a nanofluid increased the heat transfer coefficient. An increase from 0.005% to 0.02% in volume concentration increased heat transfer coefficient enhancement from 17.9% to 26.0%, respectively. Ali et al. [90] ran an experimental loop moving SiO_2_ nanofluid through a cylindrical, copper tube fitted with thermocouples at various nanoparticle concentrations. At a concentration of 0.001%, the heat transfer coefficient was enhanced by 9% relative to the base fluid alone, while at a concentration of 0.007%, there was a 27% enhancement. Therefore, as the concentration of nanoparticles increased, the heat transfer coefficient increased. Figure 18 below shows the convective heat transfer coefficient with varying axial distance (x/D) and varying concentrations of SiO_2_–water nanofluids from 0.001% to 0.007%. As observable from Figure 18, as the concentration increases, the convective heat transfer coefficient increases as well from around 2250 $W/\left(m^{2}\cdot K\right)$ to around 2600 $W/\left(m^{2}\cdot K\right)$, presenting a direct relationship.

**Figure 19:**
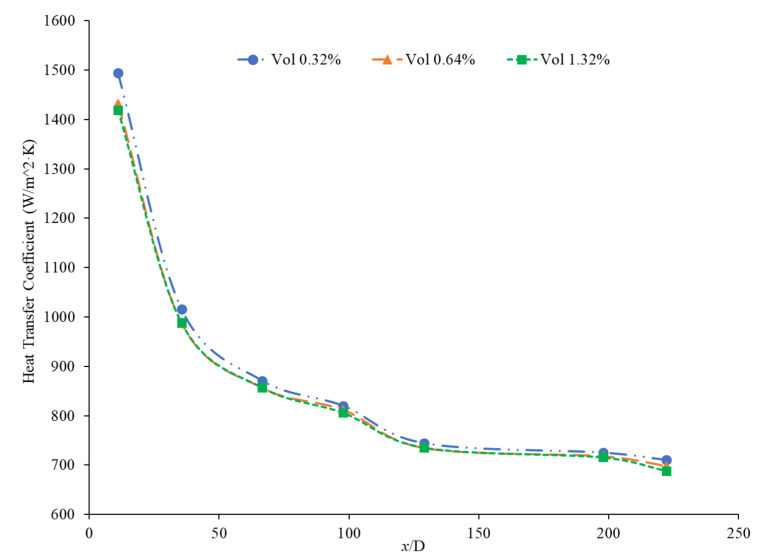
Heat transfer coefficient as a function of axial location for ZrO_2_–water nanofluids at volume concentrations of 0.32%, 0.64%, and 1.32%: Vol 0.32% [91], Vol 0.64% [91], Vol 1.32% [91].

Rea et al. [91] measured the heat transfer coefficient of Al_2_O_3_–water nanofluids at a volume fraction ranging from 0.65% to 6%. The experiments were conducted using a 1.01-m-long pipe with an internal diameter of 4.5 mm under laminar flow conditions. As the concentration of nanoparticles increased, the heat transfer coefficient of the nanofluids also increased. At a Reynolds number of 1117, the Al_2_O_3_–water nanofluid with a volume concentration of 6% had an average heat transfer coefficient of 1683.62 $W/\left(m^{2}\cdot K\right)$, while the Al_2_O_3_–water nanofluid with a volume concentration of 2.76% had an average heat transfer coefficient of 1145.80$\;W/\left(m^{2}\cdot K\right)$. The heat transfer coefficient of ZrO_2_–water nanofluids was measured as well and, unlike the Al_2_O_3_–water nanofluids, there was a decrease in the heat transfer coefficient as the concentration of nanoparticles was raised. The ZrO_2_–water nanofluids at a Reynolds number of approximately 210 had an average heat transfer coefficient of 911.5 $W/\left(m^{2}\cdot K\right)$, 891.4 $W/\left(m^{2}\cdot K\right)$, and 887.1 $W/\left(m^{2}\cdot K\right)$ for volume concentrations of 0.32%, 0.64%, and 1.32%, respectively. Figure 19 presents the heat transfer coefficient as a function of axial location for the ZrO_2_–water nanofluid at volume concentrations of 0.32%, 0.64%, and 1.32%. The decrease in heat transfer coefficient as concentration increases is caused by a reduction in the specific heat of the base fluid from the addition of nanoparticles.

**Figure 20:**
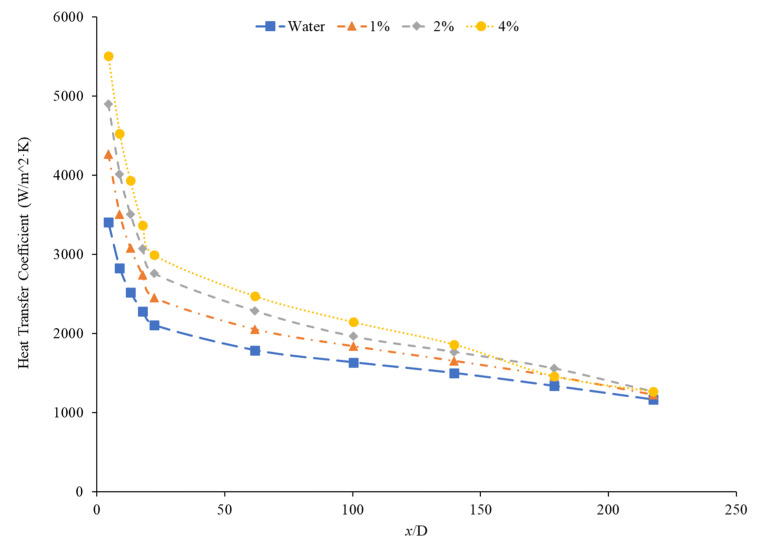
Convective heat transfer coefficient as a function of axial distance (x/D) for CuO–water-based nanofluids varying in volume concentration 1% [93], 2% [93], 4% [93].

Kai et al. [92] measured the heat transfer coefficient of SiO_2_–water nanofluids in a circular tube with a diameter of 0.8 mm under laminar flow with a Reynolds number of 112 and concentrations of 0.1–0.5 weight percent. The enhancement in the heat transfer coefficient was greatest in the nanofluid at 0.5 wt% compared to the other lower concentration nanofluids. The heat transfer coefficient also decreased along the length of the pipe for all concentrations. The heat transfer coefficient was highest at the entrance to the pipe due to the greater flow disturbance at the entrance to the pipe. Ali et al. [90] also found convective heat transfer to increase most significantly around the entrance region of a circular tube as the nanoparticle concentration increased. The maximum enhancement observed was a 19% increase from the base fluid. Umer et al. [93] noted that while running Cu_2_O–water nanofluids through a microchannel, the increase in concentration of particles led to higher heat transfer coefficient. This trend occurred despite an increased viscosity and increased boundary layer size as concentration increased. Both an increased viscosity and increased boundary layer size can decrease the heat transfer coefficient, but the increased thermal conductivity from the addition of nanoparticles had a more dominant effect, leading to an increased heat transfer coefficient. The differences among concentrations were diminished as the distance from the pipe inlet increased, indicating that the effects of nanoparticle concentration are most significant in the entrance region. As shown in Figure 20, heat transfer coefficient for the CuO nanofluid is initially higher for higher concentrations. As it progresses into the thermally developed region, the differences are less discernible.

**Figure 21:**
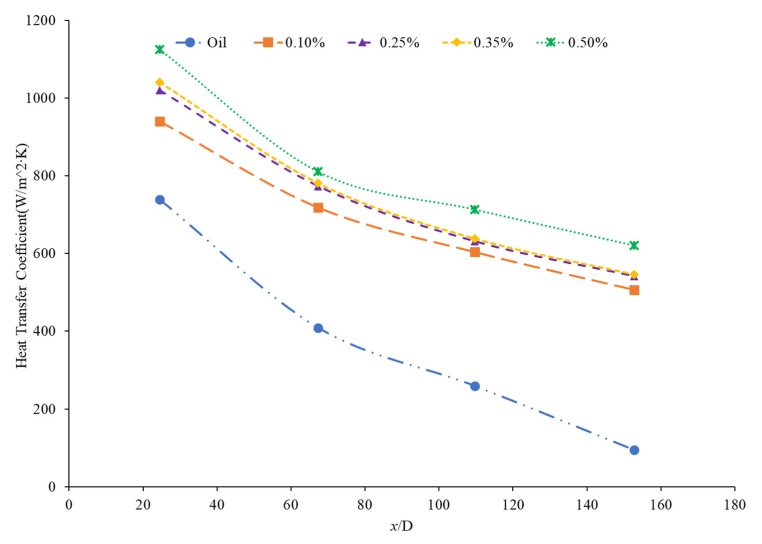
Convective heat transfer coefficient as a function of axial distance (x/D) for TiO_2_–Oil-based nanofluids varying in volume concentration: 0.5% [82], 0.35% [82], 0.25% [82], 0.10% [82], Oil [82].

Heris et al. [82] conducted oil-based nanofluids through a heated tube and reported similar findings. Higher concentrations of nanofluids resulted in higher heat transfer coefficients, but saw less enhancement as it moved further through the pipe. This trend was also observed by Minakov et al. [94], who ran a similar experiment with water-based CuO nanofluids. Higher concentrations resulted in higher heat transfer coefficients, but the enhancement decreased as it moved through the pipe further. Heris’ observations for the TiO_2_ nanofluids at different volume concentrations are shown in Figure 21.

Wen and Ding [95] measured the heat transfer coefficient of Al_2_O_3_–water nanofluids in a copper tube with a length of 970 mm and an inside diameter of 4.5 mm. The nanofluids have a volume fraction of 0.6%, 1%, and 1.6%, and the nanofluid heat transfer coefficient was measured at a Reynolds numbers of around 1050 and 1600. There was an increase in the heat transfer coefficient as the concentration of the nanofluid increased. Particle motion increases with an increase in concentration in the nanofluid, contributing to the increase in heat transfer coefficient. Additionally, the length of the region where the fluid is thermally developing increased with an increased concentration of nanofluid. Generally, increasing the concentration in a nanofluid will increase the heat transfer coefficient by increasing the thermal conductivity of the nanofluid. In some cases, an increase in viscosity and a decrease in specific heat from the addition of nanoparticles can reduce the heat transfer coefficient.

### 5.4. Effects of Nanoparticle Shape on the Heat Transfer Coefficient

**Figure 22:**
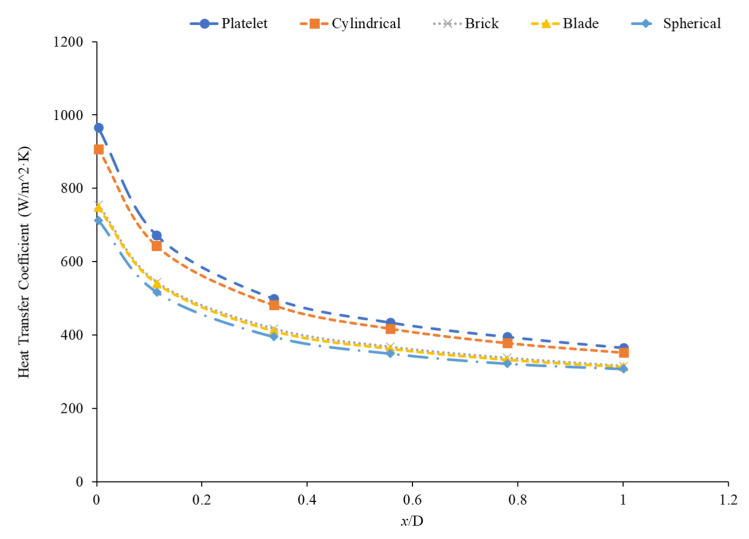
Heat transfer coefficient as a function of axial distance for varying shapes of Al_2_O_3_ water-based nanofluids: Platelet [100], Cylindrical [100], Brick [100], Blade [100], Spherical [100].

Ding et al. [81] indicated that nanoparticles with a higher aspect ratio tended to have higher heat transfer enhancement. Elias et al. [96] studied the effect of five different shaped boehmite alumina nanoparticles (cylindrical, platelets, bricks, blades, and spherical) on the heat transfer coefficient. It was concluded that the cylindrical-shaped nanoparticles presented the best heat transfer characteristics, in addition to its heat transfer rate. Elias et al. [97] calculated the heat transfer coefficient of AlOOH–50% water–50% ethylene glycol nanofluids with various nanoparticle shapes to analyze the effect of nanoparticle shape on the heat transfer coefficient. The nanoparticle shapes considered were cylinder, blade, brick, and platelet. Based on the calculations performed, the nanofluids containing cylinder-shaped nanoparticles were predicted to have the highest heat transfer coefficient. This could be explained by the cylindrical shape nanoparticles having the highest minimization of entropy generation compared to the other shapes, leading to a higher heat transfer coefficient. Chen et al. [98] measured the heat transfer coefficient of titanate nanotube–water nanofluids. The experiments were performed in a 2-m-long and 3.97-mm-diameter copper tube. The heat transfer coefficient showed a greater enhancement compared to pure water due to the thermal conductivity enhancement. One of the mechanisms attributed to the larger increase in heat transfer coefficient is the shape of the nanoparticles. The nanotubes have an aspect ratio of 10, meaning the length is 10 times greater than the nanotube diameter. This shape facilitates more particle contact than spherical particle shapes. This greater contact can lead to more energy transfer in the fluid, increasing the heat transfer coefficient. Even though there have been several studies to find that cylinder-shaped nanoparticles with the highest heat transfer coefficient create nanofluids, studies have not necessarily found that nanofluids with cylinder-shaped nanoparticles have the highest thermal conductivity [99]. It is possible that the effect of nanoparticle shape on heat transfer coefficient is more complicated than just the enhancement in thermal conductivity from the addition of the nanoparticles. Ekiciler et al. [100] performed a numerical simulation of Al_2_O_3_–water nanofluids with various nanoparticle shapes, including blade, brick, cylinder, platelet, and spherical. The simulation was conducted for flows in a triangular duct with a hydraulic diameter of 8 mm and a length of 1 m. A constant heat flux was applied in the simulation on laminar flows with a Reynolds number ranging from 100 to 500. In the simulations, the nanofluids with cylinder- and platelet-shaped nanoparticles had a similar average heat transfer coefficient that was greater than the heat transfer coefficient of the nanofluids containing brick, blade, and spherical nanoparticles. This relationship can be clearly seen in Figure 22. There is a limited amount of information available on the effect of nanoparticle shape on heat transfer coefficient, but several studies have shown that cylindrical-shaped nanoparticles produce high heat transfer coefficient nanofluids.

### 5.5. Effects of Nanoparticle Size on the Heat Transfer Coefficient

**Figure 23:**
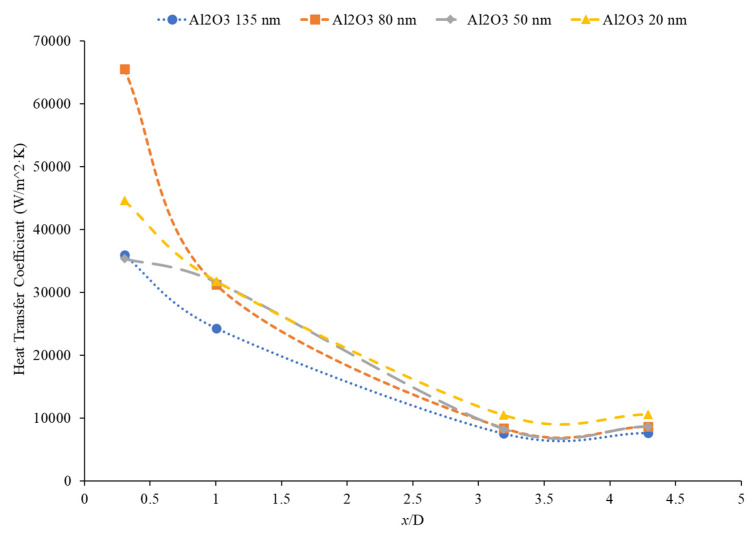
Heat transfer coefficient as a function of distance for Al_2_O_3_–water-based nanofluids with varying sizes of nanoparticles: Al_2_O_3_ 135 nm [103], Al_2_O_3_ 80 nm [103], Al_2_O_3_ 50 nm [103], Al_2_O_3_ 20 nm [103].

Norouzipour et al. [101] studied the effect of size of silicon oxide nanoparticles on the pool boiling heat transfer coefficient. These nanoparticles were mixed within a base fluid of deionized water. The sizes of particles were calculated through TEM tests. Volume concentrations ranged from 0.01% to 1%. The sizes of nanoparticles studied consisted of 11 nm, 50 nm, and 70 nm. The boiling heat transfer coefficient increased as the size of nanoparticles increased over all ranges of volume concentrations studied. Timofeeva et al. [102] investigated the effect of SiC nanoparticle size in a mixture of 50–50% mixture of ethylene glycol (EG) and water on the turbulent and laminar flow heat transfer efficiency. The nanoparticles varied from 16 nm to 90 nm. The larger nanoparticles at 90 nm showed better heat transfer properties in both flow regimes. Ali et al. [90] measured the heat transfer coefficient of SiO_2_–water nanofluids, and found that the nanofluids behave more like a typical fluid than a solid–fluid mixture. The behavior of a nanofluid is unlike that of a solid fluid mixture with micro and milli sizes. The unique features of the nanofluid contribute to the enhanced heat transfer coefficient because the nanoparticle motion will increase energy transfer and create a steeper temperature gradient, therefore enhancing the heat transfer coefficient. Anoop et al. [103] conducted experiments on nanofluid heat transfer coefficient at a constant heat flux using Al_2_O_3_–water nanofluid at a concentration of 4 wt% as well as 45 nm and 150 nm nanoparticles. At a Reynolds number of approximately 1550, the nanofluid containing 45 nm Al_2_O_3_ nanoparticles provided a greater increase in heat transfer coefficient compared to the nanofluid containing 150 nm nanoparticles. The increase in heat transfer coefficient for both nanofluids was larger than the increase in thermal conductivity of the nanofluids, suggesting that other effects, such as particle migration or thermal dispersion, contribute to the increase in heat transfer coefficient as well. Heidarshenas et al. [104] measured the heat transfer coefficient of Al_2_O_3_–water nanofluids containing nanoparticles with diameters of 20 nm, 50 nm, 80 nm, and 135 nm. The experiments were conducted using a micro channel with a length of 52 mm and a hydraulic diameter of 632 μm, and the nanofluids were at a concentration of 0.1 wt%. As the size of the nanoparticles increased, the heat transfer coefficient of the nanofluid decreased. The maximum Nusselt number increase was 21.9%, 21.1%, and 18.7% for the 20 nm, 50 nm, and 80 nm nanofluids, respectively. The nanofluids containing 135 nm nanoparticles saw a reduction in the Nusselt number compared to water, while the maximum reduction was 8.5%. The large particle may lead to agglomeration which can decrease heat transfer. The superior heat transfer by the smaller particles can be caused by a better dispersion of the particles in the base liquid and an increase in Brownian motion of the small particles. In their solar heat exchanger coolant experiment, Ebrahimnia-Bajestan et al. [73] confirmed that a smaller particle size leads to increased heat transfer characteristics due to the increase in Brownian motion over larger particles. At 2.3% concentration, the heat transfer coefficient drops from 560 $W/\left(m^{2}\cdot K\right)$ to around 490 $W/\left(m^{2}\cdot K\right)$ as the particle diameter increases from 20 to 100 nanometers. Figure 23 below shows the heat transfer coefficient as a function of distance for varying sizes of nanoparticles ranging from 20 nm to 135 nm. The 135 nm nanoparticles had the lowest heat transfer coefficient, while the 80 nm had the highest heat transfer coefficient up to around 1 3.25 x/D while the 20 nm size had the highest.

**Figure 24:**
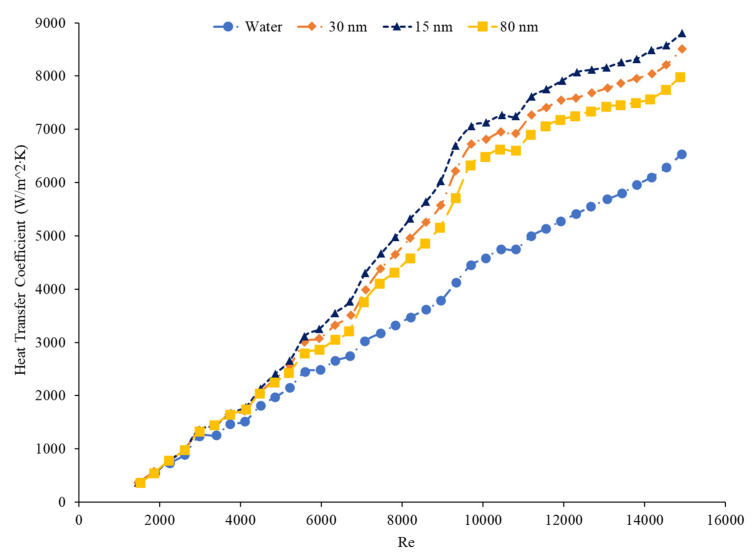
Heat transfer coefficient as a function of the Reynolds number for SiO_2_–water nanofluids with nanoparticle diameters of 15 nm, 30 nm, and 80 nm: 15 nm [105], 30 nm [105], 80 nm [105].

Zhang et al. [105] measured the heat transfer coefficient of SiO_2_–water nanofluids in a 10 mm copper tube. The SiO_2_ nanoparticles had diameters of 15 nm, 30 nm, and 80 nm. Figure 24 presents the heat transfer coefficient of the SiO_2_–water nanofluids with particle diameters of 15 nm, 30 nm, and 80 nm as a function of the Reynolds number. The highest heat transfer coefficient was in the nanofluid containing 15 nm nanoparticles, the smallest nanoparticles measured. There are several reasons for the high heat transfer coefficient in the small nanoparticles. First, since the volume concentration of the three nanofluids was equal, the nanofluid with the smallest particles had the greatest quantity of nanoparticles. Additionally, the smaller particles have a higher surface area to volume ratio. This creates more contact between particle surface and liquid for the smaller nanoparticles. Finally, there was a greater amount of random motion of particles in the nanofluid containing the smallest particles. The thermal conductivity and viscosity of the three nanofluids were measured as well. The nanofluid with 15 nm nanoparticles had both the highest thermal conductivity and viscosity. Based on the heat transfer coefficient results, the increase in thermal conductivity had a more dominant effect on the heat transfer coefficient than the viscosity. The increase in thermal conductivity, from the reducing particle size, still increased the heat transfer coefficient despite the increase in viscosity. In most cases, smaller nanoparticles will create nanofluids with a higher heat transfer coefficient due to greater particle motion and reduced particle agglomeration. 

### 5.6. Effects of Surfactant on the Heat Transfer Coefficient

**Figure 25:**
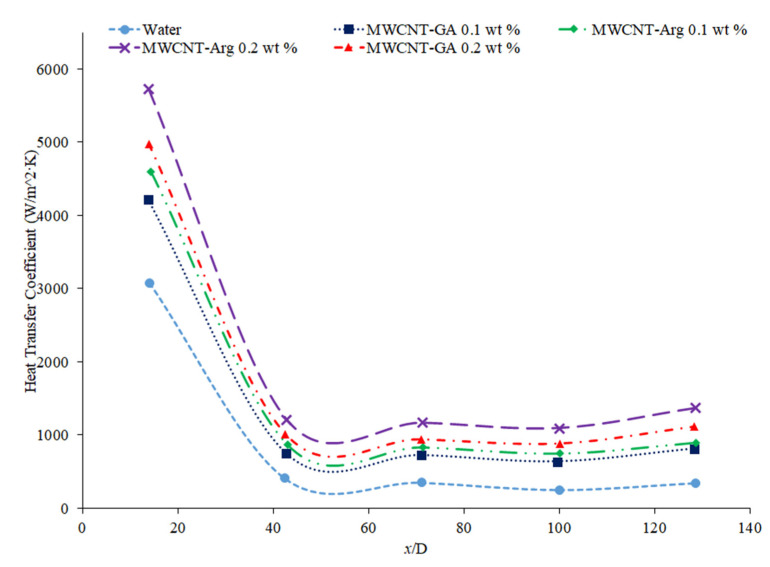
Heat transfer coefficient as a function of location for MWCNT–water nanofluids with a gum Arabic (GA) and arginine (Arg) amino acid group at a Reynolds number of 2000: MWCNT-GA 0.1 wt % [110], MWCNT-Arg 0.1 wt % [110], MWCNT-Arg 0.2 wt % [110], MWCNT-GA 0.2 wt % [110].

Utomo et al. [80] found that surfactants and organic polymers which useful in stabilizing nanofluids could decrease the heat transfer coefficient if they have a lower thermal conductivity than the base fluid. Ding et al. [81] observed in the use of SDBS surfactant for ethylene glycol-based titania and aqueous-based nano-diamond nanofluids that a foam was present, which may have contributed to poor heat transfer. Murshed and Castro [106] measured the heat transfer coefficient of TiO_2_–water nanofluids using cetyl trimethyl ammonium bromide (CTAB) as a surfactant to improve the dispersion of the nanoparticles. The experiments were conducted using a copper tube with a length of 360 mm and an inner diameter of 4.5 mm, and the nanofluids had volume concentrations of 0.2%, 0.4%, 0.6%, and 0.8%. At a Reynolds number of 1700, the heat transfer coefficient of the nanofluids increased with an increase in concentration of nanoparticles. Xuan et al. [107] measured the effect of sodium dodecyl benzene sulfonate (SDBS) surfactant on the heat transfer coefficient of Cu–water nanofluids. The nanofluids had volume fractions from 0 to 0.8% and the concentration of surfactant varied from 0 to 0.1 wt%. As the concentration of surfactant increased, the heat transfer coefficient of the nanofluids decreased. The surfactant has the potential to adhere to the heat transfer surface area as the flow is flowing which will interfere with the heat transfer to the working fluid. Halelfadl et al. [108] measured the heat transfer coefficient of MWCNT–water nanofluids at a volume fraction of 0.026% with lignin and sodium polycarboxylate as surfactants. The experiments were conducted in a 0.66-m-long stainless-steel tube with an internal diameter of 18.7 mm, and the flows had Reynolds numbers ranging from 500 to 2500. For Reynolds numbers under 700, the nanofluid with lignin as a surfactant had a higher heat transfer coefficient than the nanofluid containing sodium polycarboxylate. However, for Reynolds numbers beyond 700, there was no significant difference between the heat transfer coefficient of the two nanofluids with different surfactants. Ding et al. [109] measured the heat transfer coefficient of MWCNT–water nanofluids. Carbon nanotubes generally have aggregation in a nanofluid due to their hydrophobic properties; therefore, the addition of a surfactant is necessary. Several surfactants, such as sodium laurate (SL), gum Arabic (GA), and sodium dodecyl benzene sulfonate (SDBS), were all able to successfully stabilize the nanofluids, but SDBS failed at high temperatures. The optimum surfactant chosen for the heat transfer measurements was GA at 0.25 wt%. Hosseinipour et al. [110] also investigated the effect of surfactant on the heat transfer coefficient of MWCNT–water nanofluids. The heat transfer coefficient of MWCNT–water nanofluids with GA and MWCNT functionalized with the amino acid arginine (Arg) was measured. The MWCNT–Arg–water nanofluids had a higher heat transfer coefficient than the MWCNT–GA–water nanofluids. The effect of surfactant was more significant at a higher Reynolds number. At a Reynolds number of 800, the difference between the MWCNT–GA and MWCNT–Arg nanofluids was almost negligible. Figure 25 gives the heat transfer coefficient as a function of location for the MWCNT–Arg–water and MWCNT–GA–water nanofluids at 0.1 wt% and 0.2 wt% and a Reynolds number of 2000. From this figure, it can be seen that at both 0.1 wt% and 0.2 wt% the nanofluids with MWCNT–Arg have a higher heat transfer coefficient than the nanofluids with MWCNT–GA. Additionally, the effect of surfactant is greater at 0.2 wt%. There are two possible reasons for the greater increase in heat transfer coefficient in the arginine functionalized nanofluids than the nanofluids with GA. One possibility is that the arginine-functionalized nanofluids are more stable than the nanofluids with GA. Greater stability in a nanofluid can increase particle motion and thermal conductivity, which will contribute to a higher heat transfer coefficient. Additionally, it is possible that in the process of functionalizing the MWCNT with arginine, the nanoparticle will break apart, creating more smaller particles [110]. These smaller particles can have greater motion, increasing the heat transfer coefficient. Several studies have demonstrated that the addition of surfactant can reduce nanofluid heat transfer coefficient. Surfactants generally have a lower thermal conductivity than the base fluids; therefore, the addition of surfactants could reduce the heat transfer ability of the nanofluid. Surfactants can also adhere to nanoparticles interfering with heat transfer. Different surfactants will also have effects on the heat transfer coefficient. These variations between surfactants will vary depending on the Reynolds number of the flow.

### 5.7. Effects of Flow Regime on the Nanofluid Heat Transfer Coefficient

**Figure 26:**
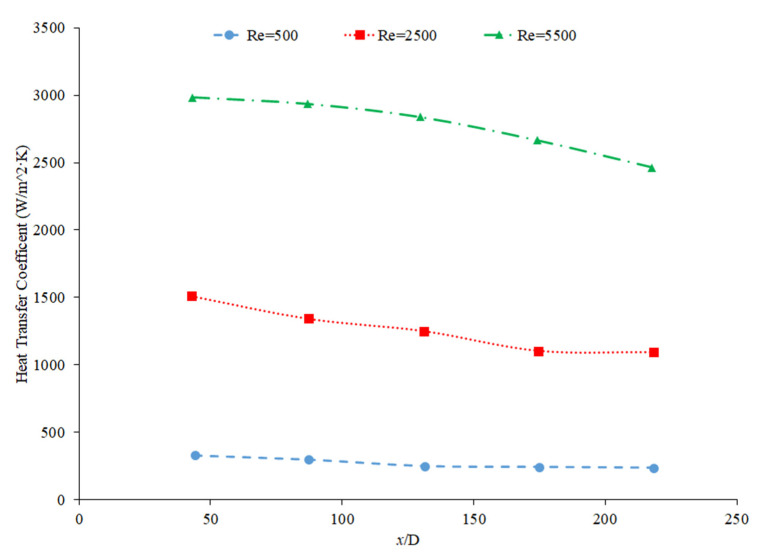
Heat transfer coefficient as a function of the nondimensionalized distance for Al_2_O_3_–kerosene nanofluids’ volume fraction of 0.05% and varying Reynolds numbers: Re = 500 [76], Re = 2500 [76], Re = 5500 [76].

**Figure 27:**
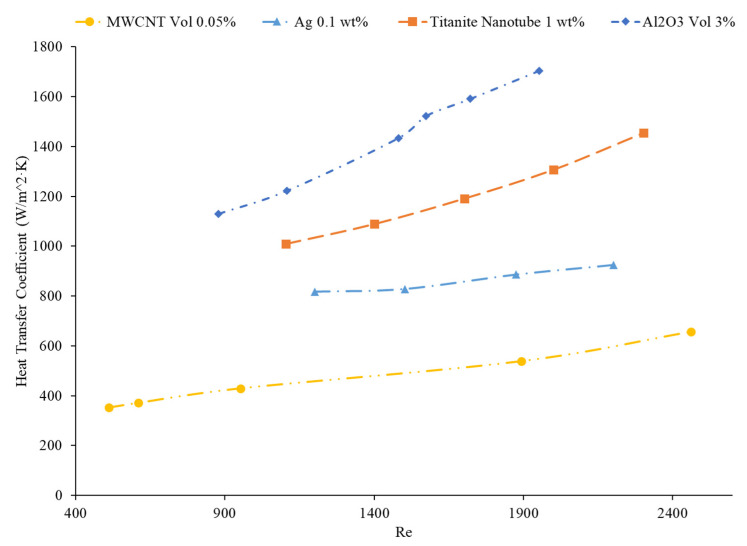
Heat transfer coefficient as a function of the Reynolds number for several water-based nanofluids: MWCNT Vol 0.05% [108], Al_2_O_3_ Vol 3% [112], Ag 0.1 wt% [116], Titanite Nanotube 1 wt% [98].

Mukherjee et al. [111] investigated the effect of the flow regime of Al_2_O_3_–water-based nanofluids on the convective heat transfer coefficient. Nanofluids at weight percent of 0.01%, 0.05%, 0.1%, 0.5%, and 1% were prepared using a two-step method. The fluid flow rate was varied from 3 lpm to 6 lpm with changing heat fluxes. The convective heat transfer coefficient increased with increasing flow rate for the nanofluids. Kim et al. [112] performed a study on the effect of flow regimes of Al_2_O_3_ water-based nanofluids at a 3% volume concentration on the enhancement of convective heat transfer coefficient. The nanofluids were tested in a circular straight tube exposed to a constant heat flux. The enhancement of the convective heat transfer coefficient increased to 15% for the laminar flow and 20% for the turbulent flow over the same increment. Therefore, a turbulent flow regime enhances the convective heat transfer coefficient of nanofluids more than a laminar flow regime. In a nanofluid radiator used for an automobile, Leong et al. [113] found that a 2% Cu–EG mixture produced an overall heat transfer coefficient of 164 $W/\left(m^{2}\cdot K\right)$ which was higher than the target coefficient of 142 $W/\left(m^{2}\cdot K\right)$ based on standard coolant. They found that while increased concentration slowed the volumetric flow rate of the nanofluid, the mass flow rate actually increased, leading to a higher heat transfer coefficient. Liu et al. [114] studied the effects of different flow regimes on the convective heat transfer coefficient. In particular, the Al_2_O_3_–water nanofluid was used with a Reynolds number varying from 600 to 4500, which covered the laminar, transition, and fully developed turbulent flow regions. The laminar and fully developed turbulent flow regions had a higher convective heat transfer coefficient than in the transition and early onset of fully developed turbulent flow regions. Ding et al. [81] also found that convective heat transfer increases as flow rate increases. However, in the case of ethylene glycol–based titania and aqueous-based nano-diamond nanofluids, it was found that the enhancement of thermal conductivity, and the convective heat transfer as a whole, deteriorates as the flow rate increases. Nguyen et al. [115] ran the Al_2_O_3_–water nanofluid through a CPU processor’s cooling system and found that the average heat transfer coefficient at a mass flow of 0.07 kg/s increased anywhere from 12% to 38%, relative to concentrations ranging from 1% to 6.8% particle concentration by volume. However, while the heat transfer coefficient increased as the flow rate increased, the overall enhancement from the based fluid was higher in laminar conditions where enhancement ranged from 14% to 42% at a mass flow of 0.03 kg/s, relative to the previously stated concentrations. For similar concentrations, Minakov et al. [94] found that higher heat transfer coefficients were achieved with higher flow rates when particle concentrations are constant. Specifically, an enhancement around 8% on average could be seen when the mass flow rate was increased from 104 g/min to 194 g/min. Sundararaj et al. [76] measured the heat transfer coefficient of Al_2_O_3_–kerosene nanofluids in the laminar, turbulent, and transitional flow regimes. The measurements were taken in a copper tube with an inner diameter of 4 mm and a length of 1 m. The heat transfer coefficient was higher in the turbulent flow regime than in the laminar flow regime. Figure 26 gives the heat transfer coefficient as a function of nondimensionalized distance for the Al_2_O_3_–kerosene nanofluids at volume fraction of 0.05% and Reynolds numbers of 500 (laminar), 2500 (transitional), and 5500 (turbulent). The increase in heat the transfer coefficient seen as the Reynolds number is increased due to a reduction in the thickness of the thermal boundary layer as the Reynolds number increases and greater particle motion in the base fluid as the Reynolds number increases. Figure 27 gives the heat transfer coefficient as a function of the Reynolds number for several water-based nanofluids. The heat transfer coefficient increases for each of the nanofluids as the Reynolds number increases. As the Reynolds number increases, the flow moves from laminar to a transitional flow regime. This will create increased random fluid motion, which will lead to an increase in the heat transfer coefficient by disrupting the formation of the thermal boundary layer in the fluid flow. In most cases, increasing the flow rate will increase the heat transfer coefficient by decreasing the thickness of the thermal boundary layer.

#### 5.7.1. Laminar Fluid Flow

**Figure 28:**
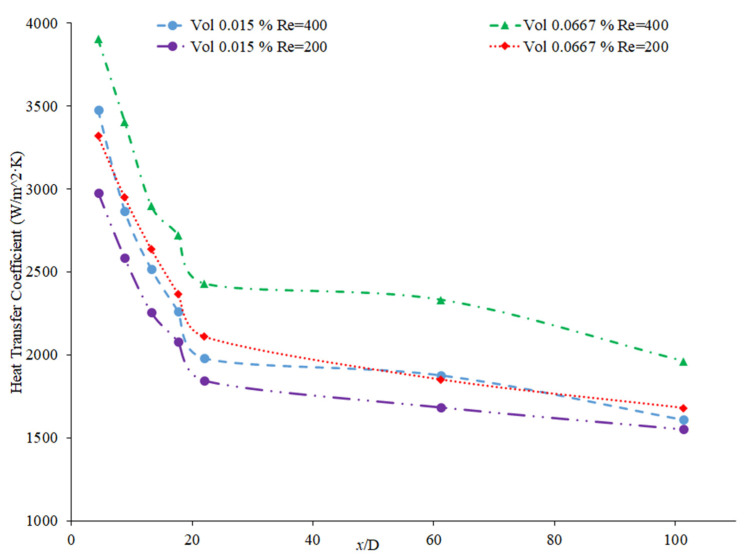
Heat transfer coefficient as a function of non-dimensionalized distance for gold–water nanofluids in laminar flow: Vol 0.015% Re=400 [118], Vol 0.0667% Re = 400 [118], Vol 0.015% Re = 200 [118], Vol 0.0667% Re = 200 [118].

Cabaleiro et al. [117] measured the heat transfer coefficient of ZnO–50% water–50% ethylene glycol nanofluids at 1 to 5 weight percent in laminar and transitional flow regimes. The experiment was conducted using a tube with a length of 2 m and an inner diameter of 8 mm. The heat transfer coefficient was found to increase with an increase in the Reynolds number. In the laminar flow regime under a constant heat flux of 5972.7 $W/m^{2}$ with Reynolds numbers of 924 and 1406, the nanofluid had an average heat transfer coefficient of 557.1 and 560.1, respectively. In the transitional flow regime with a Reynolds number of 2770 under a constant heat flux of 5972.7 $W/m^{2}$, the nanofluid had an average heat of 1574.1$\;W/m^{2}$. Vajjha et al. [83] measured the heat transfer coefficient of CuO–water nanofluid in the laminar flow regime with Reynolds numbers from 100 to 2000. The experiment was conducted in a flattened tube automobile radiator with nanofluids with a concentration of up to 6%. The heat transfer coefficient increased as the Reynolds number increased, with the maximum heat transfer coefficient at a Reynolds number of 2000 as the flow was approaching the transitional low regime. Gao et al. [58] measured the heat transfer coefficient of Fe_3_O_4_–water nanofluids in a copper tube with a length of 2.2 m and an internal diameter of 10 mm. The measurements were performed on nanofluids with volume fractions of 0.05%, 0.5%, and 2%, and laminar flows with Reynolds numbers of 400, 1200, and 2000. As the Reynolds number of the flow increased, the heat transfer coefficient of the flow increased as well. At a volume fraction of 2%, there was a heat transfer coefficient enhancement of 5.5%, 5.8%, and 6% for Reynolds numbers of 400, 1200, and 2000, respectively. The increase in micro convection in the nanofluid causes the increase in heat transfer coefficient of the nanofluid. Sabir et al. [118] measured the heat transfer coefficient of gold–water nanofluid in laminar flow. The experiments were conducted under a constant heat flux in a stainless-steel tube with a length of 580 mm and a diameter of 2.27 mm. The nanofluids were in the laminar flow regime with a Reynolds number of 200 and 400. Figure 28 gives the heat transfer coefficient as a function of nondimensionalized distance for gold nanofluids with volume fractions of 0.015% and 0.0667% at Reynolds numbers of 200 and 400. It is clear that both concentration and the Reynolds number have an impact on heat transfer coefficient. The nanofluid at a volume fraction of 0.0667% and a Reynolds number of 400 has the highest heat transfer coefficient, while the nanofluid at a volume fraction of 0.015% and a Reynolds number of 200 has the lowest heat transfer coefficient. The nanofluid at a volume fraction of 0.015% with a Reynolds number of 400 and the nanofluid at a volume fraction of 0.0667% with a Reynolds number of 200 have very similar heat transfer coefficients. An increase in the Reynolds number can increase the heat transfer coefficient by increasing the random motion of the fluid flow, while an increase in the volume fraction of the nanofluid can increase the heat transfer coefficient by increasing the thermal conductivity of the nanofluid. Therefore, the nanofluid with both a high concentration and a high Reynolds number had the highest heat transfer coefficient, while the nanofluid with the lowest concentration and Reynolds number had the lowest heat transfer coefficient. The nanofluid with a high concentration and a low Reynolds number and the nanofluid with a low concentration and a high Reynolds number were very similar, demonstrating that it is not clear which effect is more dominant. An increase in concentration or Reynolds number both have the potential to increase the heat transfer coefficient. All nanofluids showed a dramatic decrease in the heat transfer coefficient as the axial location increased, with the highest heat transfer coefficient measurements being found in the entrance region of the pipe. The high heat transfer coefficient in the entrance region could be caused by the extended thermal boundary region and the increased particle motion in the entrance region. A significant amount of research has shown that, in the laminar flow regime, the nanofluid heat transfer coefficient increases with an increase in the Reynolds number. The increase in the Reynolds number leads to more ransom motion in the fluid flow, facilitating energy transfer.

#### 5.7.2. Turbulent Fluid Flow

**Figure 29:**
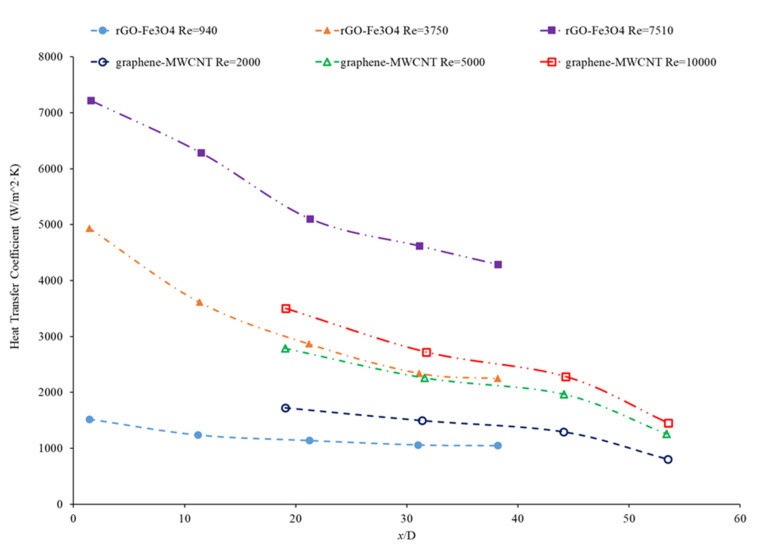
Heat transfer coefficient as function of nondimensionalized distance for various nanofluids at a volume fraction of 0.02% and a varying Reynolds number: graphene-MWCNT Re = 2000 [72], graphene-MWCNT Re = 5000 [72], graphene-MWCNT Re = 10,000 [72], rGO-Fe_3_O_4_ Re = 940 [121], rGO-Fe_3_O_4_ Re = 3750 [121], rGO-Fe_3_O_4_ Re = 7510 [121].

**Figure 30:**
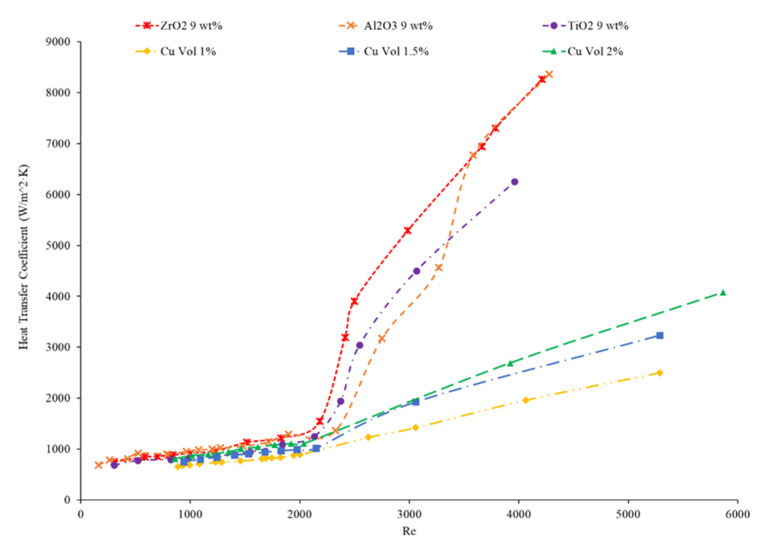
Heat transfer coefficient as a function of Reynolds number for several water-based nanofluids: ZrO_2_ 9 wt% [122], Al_2_O_3_ 9 wt% [122], TiO_2_ 9 wt% [122], Cu Vol 1% [123], Cu Vol 1.5% [123], Cu Vol 2% [123].

Baby and Sundara [119] studied the heat transfer coefficient of water-based copper oxide decorated graphene (CuO/HEG) nanofluids in the turbulent flow regime in a stainless-steel tube with a length of 1.08 m and an inside diameter of 23 mm. The nanofluids used in the experimental work had volume fractions of 0.005% and 0.01% and were tested at Reynolds numbers of 4500, 8700, and 15,500. As the Reynold number increased, the heat transfer coefficient increased as well. For the nanofluids at a volume fraction of 0.01%, the average heat transfer coefficient across the length of the tube was around 1413 at a Reynolds number of 15,500, while at a Reynolds number of 4500, the average heat transfer coefficient was only around 542. Brownian motion and particle migration both contribute to the increase in heat transfer coefficient of the nanofluid. Ahmed et al. [120] measured the heat transfer coefficient of ZnO–TiO_2_–water hybrid nanofluids in the turbulent flow regime. The nanofluids had concentrations ranging from 0.25 wt% to 0.1 wt% and the Reynolds number of the flow ranged from 5849 to 24,544. At each concentration as the Reynolds number increased, the heat transfer coefficient increased as well. Ali et al. [90] measured the heat transfer coefficient of SiO_2_–water nanofluids with concentrations of 0.001%, 0.003%, and 0.007% in a 1-m-long copper tube with an internal diameter of 16.5 mm. The fluid flows were turbulent with Reynolds numbers ranging from 8000 to 20,000. For the nanofluids at each concentration, as the Reynolds number increased, the heat transfer coefficient increased as well. Additionally, there was a correlation where the nanofluids with a higher concentration had a higher heat transfer coefficient. As the Reynolds number and the difference between the nanofluids with different concentrations both increased, the effect of concentration increased, as. Barai et al. [121] measured the effect of turbulent flow on the heat transfer coefficient of reduced graphene oxide Fe_3_O_4_–water nanofluids. The experiments were conducted in a 1-m-long and 2.54-cm-inner-diameter copper tube with nanofluids with a volume fraction of 0.01% and 0.02% and the flows had a Reynolds number varying from 940 to 7510. As the Reynolds number increased, the heat transfer coefficient increased. The heat transfer coefficient was higher in the turbulent flow regime than in the laminar flow regime than in the laminar flow regime. In Figure 29, the heat transfer coefficient, as a function of nondimensionalized distance, is given for water-based reduced graphene oxide rGO–Fe_3_O_4_ and graphene–MWCNT nanofluids with a varying Reynolds number. It can be seen that, for both nanofluids, as the Reynolds number increases, the heat transfer coefficient increases as well. The nanofluid flows in the turbulent flow regime have the highest heat transfer coefficient. The increased Reynolds number means there is more random motion in the fluid and the nanoparticles which will disrupt the formation of the thermal boundary layer, leading to an increase in the heat transfer coefficient. It is also demonstrated that the rGO–Fe_3_O_4_ nanofluid has a higher heat transfer coefficient despite having a maximum Reynolds number of 7510 compared to a maximum Reynolds number of 10,000 for the graphene–MWCNT nanofluid. Therefore, other characteristics of the nanofluid and channel characteristics will affect the heat transfer coefficient in addition to the Reynolds number. Figure 30 demonstrates the heat transfer coefficient as a function of Reynolds number for several water-based nanofluids. For each of the nanofluids, as the Reynolds number increases, the heat transfer coefficient increases as well. As the Reynolds number increases, the flow becomes more turbulent which will create more random motion in the fluid. This random motion will disrupt the development of the thermal boundary layer and create more particle motion, increasing the energy transfer in the fluid. The heat transfer coefficient increases significantly beyond a Reynolds number of 2000. Generally, at a Reynolds number of 2300, fluid flow moves from laminar to transitional flow, leading to a significant increase in random motion in the fluid. Therefore, the heat transfer coefficient will increase significantly when moving to the transitional flow. In the turbulent flow regime, increasing the Reynolds number will increase the heat transfer coefficient by reducing the thickness of the thermal boundary layer and increasing random motion in the fluid flow. The heat transfer coefficient increases significantly as the flow moves from laminar to turbulent flow.

### 5.8. Effects of Channel Characteristics on the Heat Transfer Coefficient

**Figure 31:**
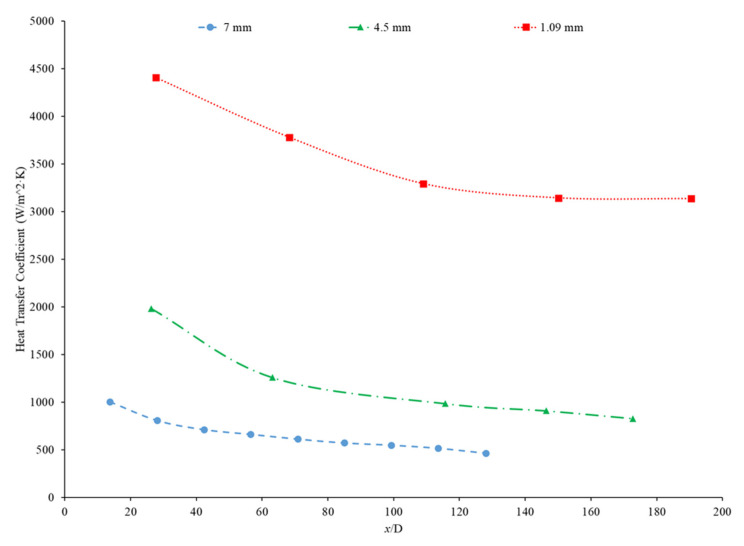
Heat transfer coefficient as a function of axial location with Al_2_O_3_–water nanofluids at a volume fraction of 1% and Reynolds numbers from 1100 to 1300 in different tube diameters: 7 mm [88], 1.09 mm [95], 4.5 mm [114].

**Figure 32:**
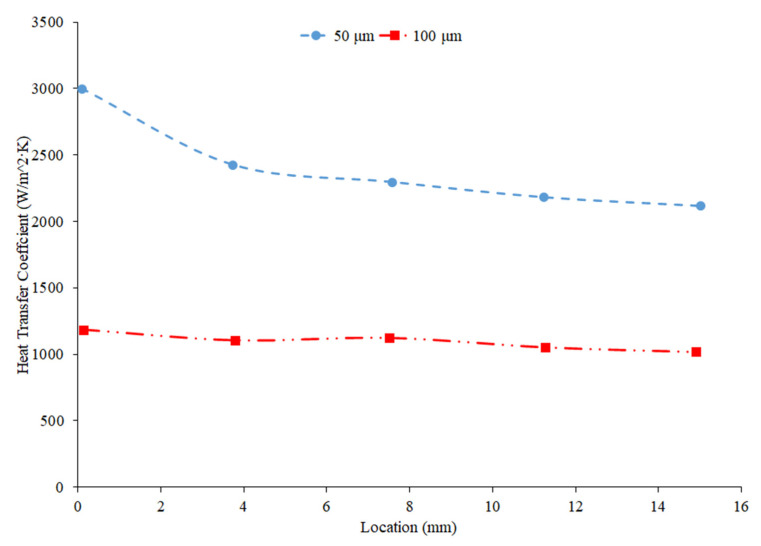
Heat transfer coefficient as a function of axial location with Al_2_O_3_–water nanofluids at 1.8% volume fraction with Reynolds numbers between 60 and 80 in square tubes with hydraulic diameters of 50 μm and 100 μm: 50 μm [125], 100 μm [125].

**Figure 33:**
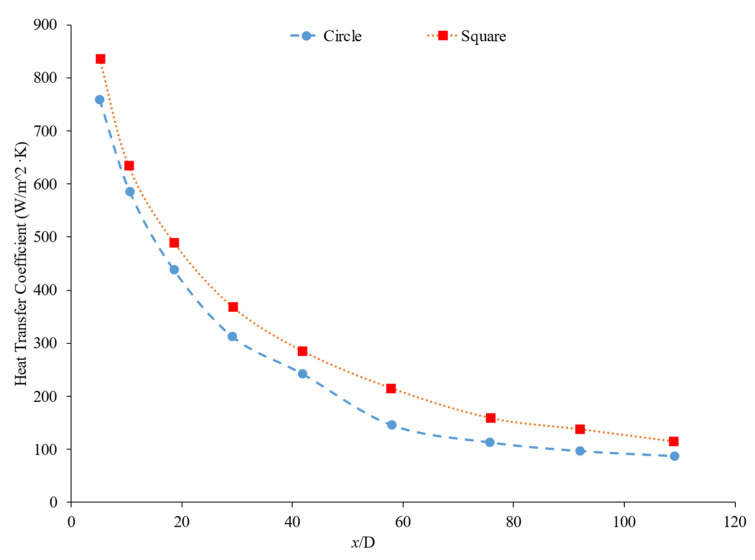
Local heat transfer coefficient of SiO_2_–water nanofluids at volume fraction of 0.07% and a Reynolds number of 621 with different shaped channels with an 8 mm hydraulic diameter: Circle [130], Square [130].

Channel characteristics also play a role on the heat transfer coefficients. Kumaresan et al. [124] looked at the effect of multi-wall carbon nanotubes (MWCNT) in a 70% water and 30% ethylene glycol mixture. In particular, these nanotubes are placed in a tubular heat exchanger with varying lengths. There was an enhancement in the convective heat transfer coefficient for a shorter length. This held true because of the movement of the carbon nanotubes. This movement of the carbon nanotubes limits the thermal boundary layer from growing at a faster rate. Ebrahimnia-Bajestan et al. [73] measured the heat transfer coefficient of TiO_2_–water nanofluids under a constant heat flux. The experiments were conducted in a 2-m-long tube with a 7.8 mm inner diameter. The nanofluids tested were at volume fractions up to 2.3%. The effect of concentration was large at low axial distance, but at longer axial distances, the effect of the different concentrations was negligible. Therefore, there is potentially a greater effect of nanoparticle concentration in shorter length tubes. Jung et al. [125] measured the heat transfer coefficient of Al_2_O_3_ nanofluids with a base fluid of water and 50% water–50% ethylene glycol in various sizes of microchannel. The nanofluids with a base fluid of 50% water and 50% ethylene glycol were unable to establish a steady flow due to their high viscosity in the 50 μm by 50 μm and 50 μm by 100 μm channels. The Al_2_O_3_–50% water–50% ethylene glycol nanofluid was however able to establish a steady flow in the 100 μm by 100 μm channel. Therefore, high-viscosity nanofluids are not suitable for microchannels of a small enough size. For the water-based nanofluids, the entrance region effect was greater in the 100 μm by 100 μm channel than it was in the 50 μm by 50 μm channel. Kim et al. [126] measured the heat transfer coefficient of Al_2_O_3_–water nanofluids in different diameter tubes. The tubes all had a length of 0.76 m and the inside diameters used were 0.8, 1.6, and 2.0 mm. The nanofluids tested had concentrations of 0.5, 1.0 and 2.0 weight percent. The average heat transfer coefficient increased as the diameter of the tube decreased. The effect of decreasing the size of the tube was also larger for flows at a higher Reynolds number. Utomo et al. [80] measured the heat coefficient of Al_2_O_3_–water nanofluids in tubes with a diameter of 4.57 mm and 10 mm. With the nanofluids, a concentration of 9 wt% and a Reynolds number of 1070 the nanofluids in the 4.57 mm tube had a higher heat transfer coefficient than the nanofluids in the 10 mm tube. In Figure 31, the heat transfer coefficient of Al_2_O_3_–water nanofluids at 1% volume fractions and Reynolds number of about 1100–1300 is given. The nanofluid heat transfer coefficient was measured in tubes with an inner diameter of 7 mm, 4.5 mm, and 1.09 mm. It can be seen that the nanofluid in the 1.09 mm tube had a significantly higher heat transfer coefficient. Several researchers have concluded that as axial distances increase nanofluids approach a constant Nusselt number around 4.36 [127,128,129]. The thermal conductivity of the three fluids should be approximately equal since the nanofluid condition is the same; therefore, the smaller tube diameter must correspond to a higher heat transfer coefficient since either an increase in heat transfer coefficient or a decrease in tube diameter will lead to a higher heat transfer coefficient at a constant Nusselt number. In Figure 32, the heat transfer coefficient as a function of axial location for Al_2_O_3_–water nanofluids is given for square tubes with different hydraulic diameters. The same relationship between the tube diameter and heat transfer is shown where the nanofluids with a hydraulic diameter of 50 μm are higher than the tube with a hydraulic diameter of 100 μm. The heat transfer coefficient of the smaller tube is about twice as large as the heat transfer coefficient of the tube that is twice as large. This further suggests that the heat transfer coefficient is inversely proportional to the tube diameter. Additionally, as the size of the square decreases, the perimeter to area ratio of the channel cross section increases. This means there is creator contact between the fluid and the channel surface. This will lead to greater heat transfer surface area per fluid volume. Pourfayaz et al. [130] measured the heat transfer coefficient of SiO_2_–water nanofluids in square- and circle-shaped channels. Both channels had a hydraulic diameter of 8 mm and the nanofluids were subjected to a constant heat flux. In each measurement conducted for the same nanofluid conditions and flow conditions, the square tube had a greater heat transfer coefficient. Figure 33 gives the heat transfer coefficient as a function of axial distance for the SiO_2_–water nanofluids at a concentration of 0.07% and a Reynolds number of 621. The square channel has a greater perimeter than the square channel for the same hydraulic diameter. Therefore, there is greater contact between the nanofluid and the channel wall in the square channel. This provided a greater heat transfer surface area, which will give the square tube a greater heat transfer coefficient. Smaller tubes and square tubes generally have a higher heat transfer coefficient since they have a higher perimeter to cross sectional area ratio, creating more contact between the fluid and the wall. Shorter tubes also have a higher average heat transfer coefficient than longer tubes since the highest heat transfer coefficient is in the entrance region. In shorter tubes, a greater portion of the flow is in the entrance region.

### 5.9. Effect of Specific Heat of Heat Transfer Coefficient

The addition of nanoparticles will also affect the specific heat of a nanofluid which can also affect the heat transfer properties. Since the specific heat of the materials commonly used in nanoparticles is typically lower than the base liquid, in most cases, the addition of nanoparticles will reduce the overall specific heat of the nanofluid [131]. Unlike other nanofluid properties, the only factors that will affect the specific heat is the material and the concentration of the particles. Particle characteristics, such as size and shape, do not affect the specific heat [132]. Bergman [133] analyzed the effect of specific heat on the heat transfer effectiveness of nanofluids as heat transfer fluids in the laminar flow regime. It was considered important to examine the effect of nanoparticles on both thermal conductivity and specific heat. High thermal conductivity from the addition of nanoparticles can increase heat transfer in internal convection by allowing greater energy transfer between the wall and fluid. A reduction in the specific heat from nanoparticles can potentially reduce heat transfer effectiveness since the bulk fluid temperature will increase more easily. This can lead to an increase in the temperature of the surface being cooled since the fluid is now at a higher temperature as well. It was determined that nanofluids are most effective in short tubes and high flow rates for internal convection. In these cases, the nanofluid temperature is not allowed to increase significantly. This allows the benefits of high thermal conductivity to be utilized, without a significant on the reduced specific heat. Asadi [134] proposed that the specific heat of the nanofluid does not affect the heat transfer coefficient nanofluid in fully developed laminar flow. In fully developed laminar flow, the Nusselt number is a constant so only the thermal conductivity affects the heat transfer coefficient. This was supported by measurements for heat transfer coefficient of thermal oil-based nanofluids where, in all cases, an increase in concentration, which also increases thermal conductivity, led to an increase in heat transfer performance compared to the base fluid. In turbulent flow, the specific heat can influence the heat transfer coefficient. For turbulent flow, the heat transfer performance of the thermal oil nanofluids compared to the base fluids increased or decreased, depending on the conditions. Vajjha et al. [135] measured the heat transfer coefficient of 60% ethylene–40% water-based CuO, Al_2_O_3_, and SiO_2_ nanofluids. Of the three nanofluids, the CuO nanofluid had the lowest specific heat, but also had the highest heat transfer coefficient. The CuO nanoparticle also had the highest thermal conductivity of nanoparticles considered. This indicates that the effect of the thermal conductivity of the nanoparticles may be more significant than specific heat.

## 6. Experimental Setup

**Figure 34:**
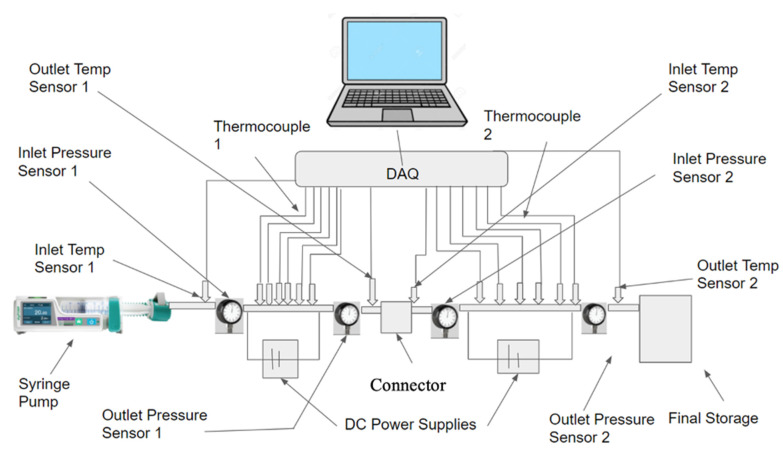
Test assembly.

The purpose of this paper is to investigate the heat transfer effects of a connector between two microchannels carrying nanofluid. The experimental setup designed for this investigation is shown in Figure 34. The setup is a linear non-circulating design where the fluid passes through the experimental setup only once before being collected in a reservoir. Fluid was pumped through the system using a New Era Pump Systems’ NE-1000 syringe pump fitted with a Hamilton 100 mL syringe. The pump was connected to the microchannel assembly via Hamilton 86510 plastic tubing. This tubing leads into an Upchurch Scientific 5700184 four-way junction. The tubing was connected with an adapter to one junction port and the first microchannel was connected via an adapter to the port opposite to the tubing. This junction also houses a pair of sensors, one in each of the unused ports, that records the fluid’s temperature and pressure. The fluid was carried through an adapter from the junction into the microchannel assembly. The microchannel assembly consists of two stainless steel microchannels, each with an inner diameter of 210 μm and an outer diameter of 413 μm. The first microchannel was connected to the second microchannel with a stainless-steel connector section, which contained the intermediate storage gap. This second microchannel then ended with another four-way junction which served as the outlet. Another section of the Hamilton 86510 plastic tubing was used to carry the fluid into a waste storage container. To keep the junctions from leaking, so as not to disrupt the fluid flow, Loc-Tite epoxy was used to seal the connections.

### 6.1. Heating System

Each of the microchannels were independently resistively heated. A Sorensen XPH 20-20 DC power supply was connected by wire directly to the stainless steel microchannels and current is run through them. The resistivity of the steel results in heating. The initial and final sections of each microchannel were left unheated by attaching the wires 3.5 cm from each end, which limited the current to flowing in the central test section. This delayed the thermal development of the fluid until the flow was fully developed. The power supply could be set in the range of 0–20 V and 0–20 A, depending on the needed heating conditions. The resistance in each microchannel was measured via a National Instruments USB-4065 Digital Multimeter. These measurements can be used in conjunction with the Law of Conservation of Energy to determine the heat resistively generated in the microchannel, and to adjust it accordingly.

### 6.2. Temperature Measurement

A total of 23 temperature sensors were used in the assembly to record the thermal conditions in the test assembly. Furthermore, 10 RS Pro 397-1589 thermocouples, each with a diameter of 86 μm, were attached to each of the microchannels. The thermocouples were attached every 1.22 cm using thermally conductive Cotronics Duralco 132 epoxy. Once the thermocouples were attached, each microchannel was covered with 3M scotch-weld 2214 epoxy glue, which prevents heat loss from the test section. To further improve the heat retention of the assembly, each microchannel section was layered between two pieces of 1 cm dry insulation. These thermocouples have an error report of ±1.5 °C.

### 6.3. Pressure Measurement

Pressure was measured at the inlet and the outlet of each of the test assemblies. The two sensors were Omega PX26-100GV pressure transducers. Each one was powered by an Omega PST 4130 power supply with a current of 150 mA and a direct current output of 12 V. These pressure sensors have an error report of ±1%.

### 6.4. Data Acquisition Instrumentation

A National Instruments NI cDAQ-9178 base was used to record the data from the system. The thermocouples were connected to two National Instruments NI 9213 cards, with each microchannel using a separate card for its respective thermocouples. The pressure transducers were connected using a National Instruments NI 9218 card with NI9982 adapters, and the voltage drop over the microchannel assembly was recorded using a National Instruments NI 9221 card. All data from the DAQ system were run through and recorded by a National Instruments LabVIEW program. The logged data were then converted into spreadsheet form, which allowed for easier organization and analysis.

### 6.5. Nanofluid Preparation and Related Calculations

This experiment uses a variety of nanofluids, all utilizing a base of deionized water. The Fe_3_O_4_ nanofluid was created by mixing commercially purchased Fe_3_O_4_ nanoparticles into a base of deionized water. The SnO_2_ nanofluid utilized in this experiment was created from previously prepared SnO_2_ nanoparticles. These SnO_2_ nanoparticles were then mixed with deionized water without surfactants in order to create a 1 wt% SnO_2_ nanofluid. This mixture was stirred at room temperature for 24 h. After this time, in order to ensure that the fluid was fully homogenized and no precipitate was formed, the nanofluid container was sonicated for 10 min before any nanofluid was drawn off and put into the system.

## 7. Materials and Methods—Nanofluid Preparation and Related Calculations

The water–Fe_3_O_4_ nanofluids were produced by mixing water and 15–20 nm Fe_3_O_4_ nanoparticles, purchased from US Research Nanomaterials, Inc, such that the mass fraction of nanofluid was 0.01. Before each test, the nanofluid was homogenized with an ultrasonic bath to combat precipitation which occurred in the interim between tests. The nanoparticles were found to be stable with the base liquid, and no precipitation was seen for a sufficient amount of time. Before each test, the syringe pump syringe was cleaned by loading deionized water into the syringe and running the pump. This forced the water through the microchannel to clean off any sediment which built up within the microchannel and tubing. Once the assembly was cleaned, the syringe was emptied of deionized water and filled with nanofluid. The system was then run with the nanofluid. The flow rate was at first set to a low magnitude in each test to yield a low Reynolds number. The flow rate was then slowly raised to reach the required flow rate for the test. The test apparatus was flushed with deionized water after every test run. Density, specific heat, and volume fraction of nanofluids can be calculated using Equations (1)–(3), respectively [33].

(1) $$\rho_{nf}=\phi \rho_{p}+\left(1-\phi \right)\rho_{bf},$$

(2) $${\left(c_{p}\right)}_{nf}=\frac{\phi {\left(\rho c_{p}\right)}_{p}+\left(1-\phi \right){\left(\rho c_{p}\right)}_{bf}}{\rho_{nf}},$$

(3) $$\phi =\frac{w\rho_{bf}}{\rho_{p}\left(1-w\right)+w\rho_{bf}},$$

The physical properties of nanofluids including viscosity, specific heat, and density were obtained, using reference [33].

Thermocouples were used to find the outlet and inlet. These temperatures, the specific heat, the mass flow rate, the rate of heat flux, and the rate of energy absorption of the nanofluid can be found from Equations (4) and (5), respectively. The physical properties of nanofluids used are given in Table 1.

(4) $${\dot{Q}}_{absorbed}=\dot{m}c_{p}\Delta T=\dot{m}c_{p}\left(T_{out}-T_{in}\right),$$

(5) $$\dot{q}=\frac{{\dot{Q}}_{absorbed}}{A},$$

where $\dot{q}$ is the heat flux and *A* is the area used for heat transfer. If the inlet temperature is known, then the fluid temperature as a function of the distance in the pipe can be found with Equation (6).

(6) $$T\left(x\right)=T_{in}+\frac{\dot{q}\pi D}{c_{p}\dot{m}}x,$$

The surface temperature distribution as a function of distance was determined by attaching thermocouples to the outer surface of the microchannel. With the distribution of temperature along the microchannel known, the heat transfer coefficient can be determined from Equation (7).

(7) $$h\left(x\right)=\frac{\dot{q}}{\left(T_{s}\left(x\right)-T_{f}\left(x\right)\right)},$$

The temperatures were measured strictly when the fluid flow had reached steady state conditions. The heat transfer coefficient as a function of location was found for a given condition. The standard deviations of the calculated heat transfer coefficients were also found. It was seen that, for any given point, the standard deviation was small, relative to the value of the heat transfer coefficient. Thus, the error bars were ignored for this experiment.

## 8. Results and Discussion

**Figure 35:**
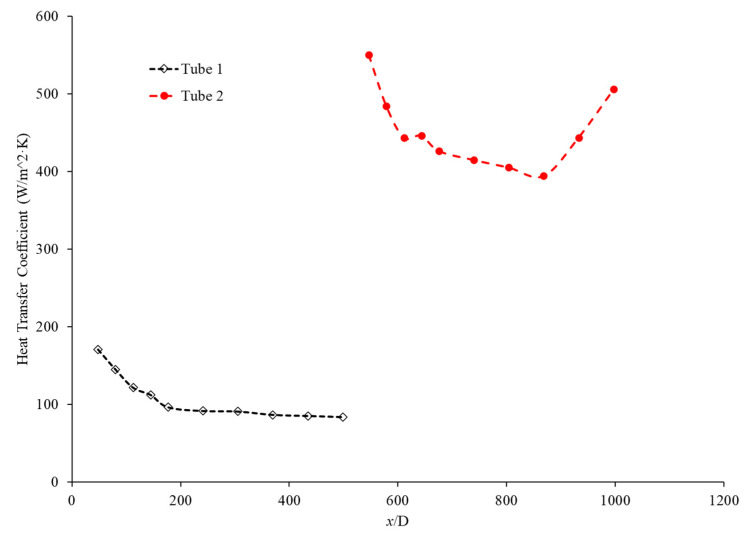
Variation of heat transfer coefficient as a function of distance for deionized water. The Reynolds number inside the microchannel was 100.

**Figure 36:**
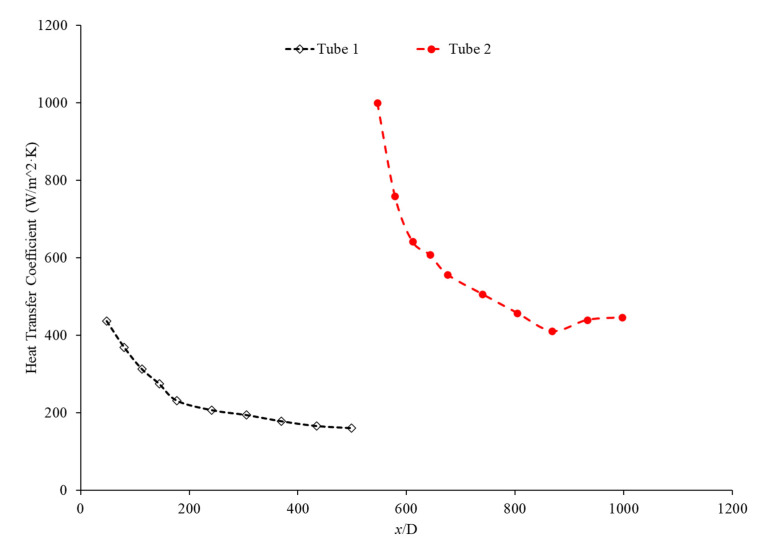
Variation of heat transfer coefficient as a function of distance for the deionized water. The Reynolds number inside the microchannel was 200.

**Figure 37:**
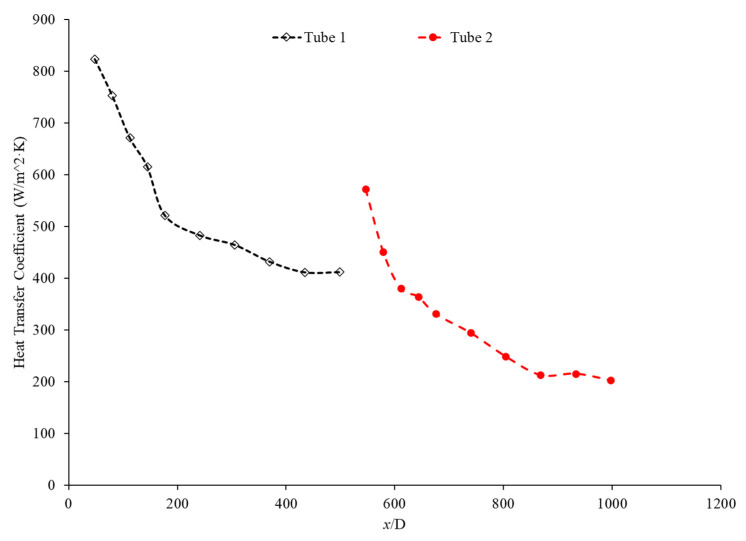
Variation of heat transfer coefficient as a function of distance for the deionized water. The Reynolds number inside the microchannel was 300.

**Figure 38:**
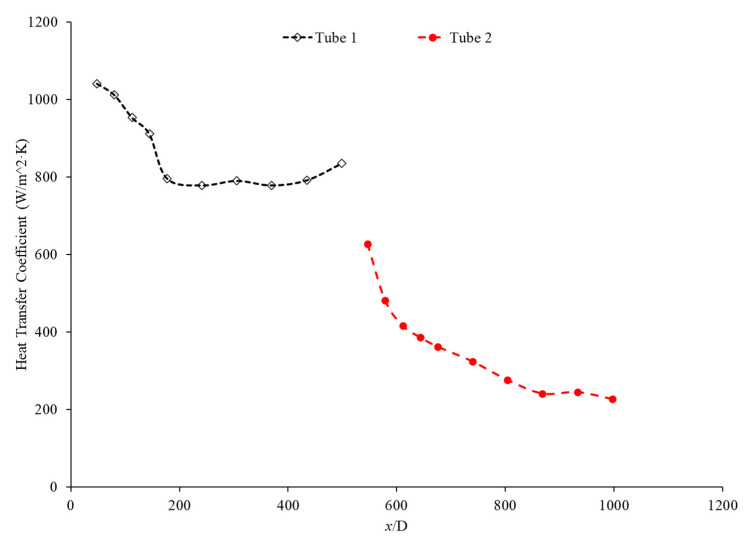
Variation of heat transfer coefficient as a function of distance for the deionized water. The Reynolds number inside the microchannel was 500.

**Figure 39:**
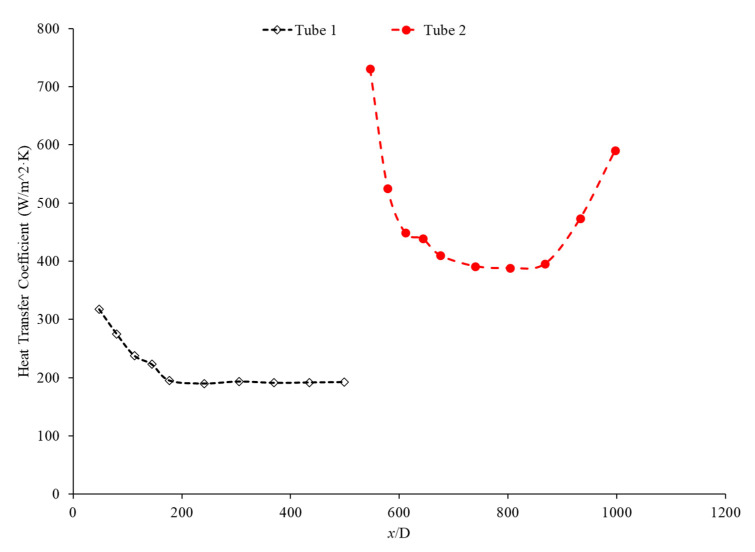
Variation of the heat transfer coefficient as a function of distance for the deionized water–Fe_3_O_4_ nanofluids. Nanofluid concentration was 1 wt%. The Reynolds number inside the microchannel was 114.

**Figure 40:**
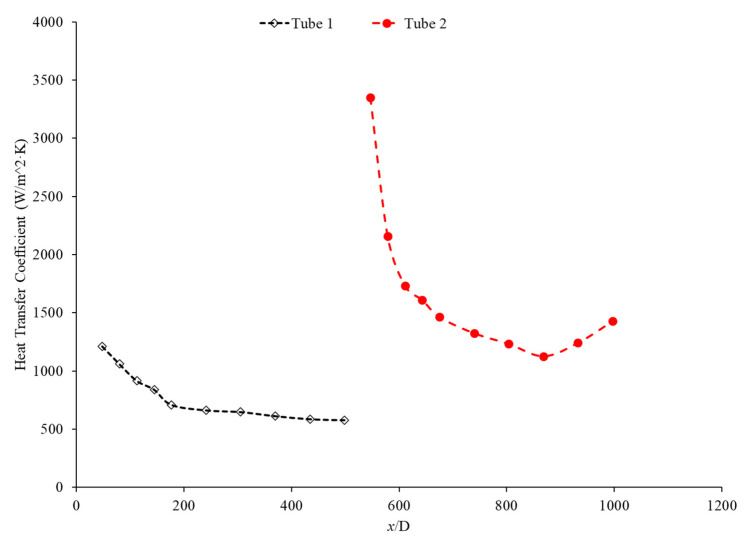
Variation of the heat transfer coefficient as a function of distance for the deionized water–Fe_3_O_4_ nanofluids. Nanofluid concentration was 1 wt%. The Reynolds number inside the microchannel was 236.

**Figure 41:**
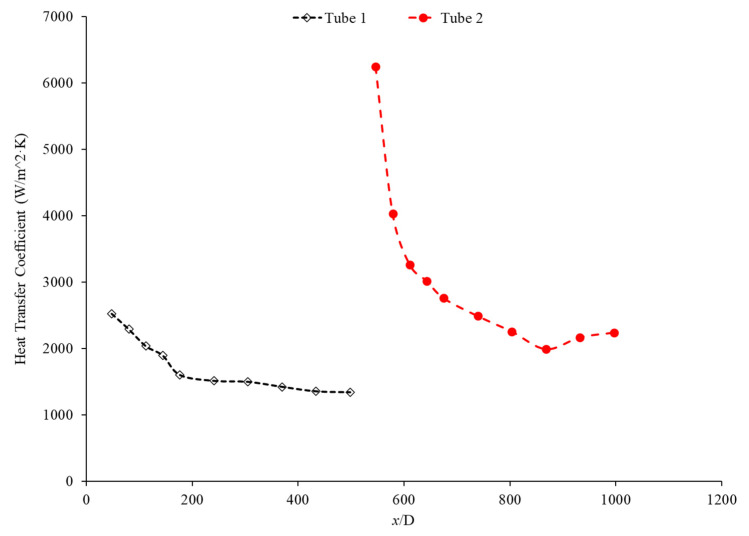
Variation of the heat transfer coefficient as a function of distance for deionized water–Fe_3_O_4_ nanofluids. Nanofluid concentration was 1 wt%. The Reynolds number inside the microchannel was 347.

**Figure 42:**
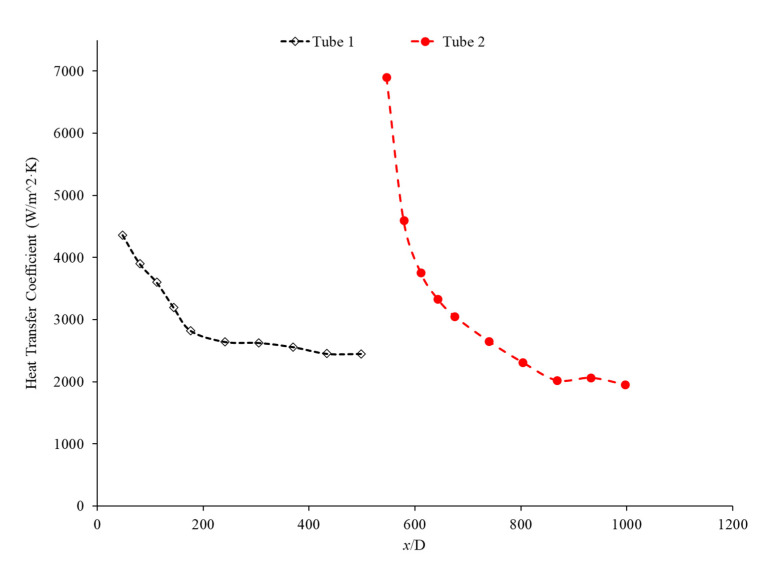
Variation of the heat transfer coefficient as a function of distance for the deionized water–Fe_3_O_4_ nanofluids. Nanofluid concentration was 1 wt%. The Reynolds number inside the microchannel was 524.

The forced convection heat transfer coefficient was measured as a function of distance for deionized water and deionized water–Fe_3_O_4_ nanofluids. The concentration of the water–Fe_3_O_4_ nanofluids was 1 wt% and the range of the Reynolds number inside the microchannel was 100–550. Figure 35, Figure 36, Figure 37 and Figure 38 show the heat transfer coefficient as a function of distance for deionized water for Re = 100, 200, 300, and 500, respectively. Error bars are visible in Figure 35. Since the error bars are small, they are removed in the rest of the figures. Figure 35 shows that the heat transfer coefficient decreases in the first channel smoothly as *x* increases; however, in the second channel, the heat transfer coefficient decreases and then increases as *x* increases from the entering point. It is thought that the fluid would “lose its memory” inside the connector, before entering the second channel. Therefore, the heat transfer coefficient starts from maximum and decreases as *x* increases again, and then it starts increasing again as *x* increases further. A similar trend was observed in the case of water–Fe_3_O_4_ nanofluids (1 wt%), when the Reynolds number was in the range of 100–250 (Figure 39 and Figure 40). In case of a low Reynolds number, the fluid temperature increases at the end of the second channel. As a result, the viscosity of working fluid decreases; therefore, the random motion of molecules and particles increases and consequently the heat transfer coefficient increases. The effects of this phenomenon become weak as the Reynolds number increases further. Figure 36 shows that the heat transfer coefficient decreases in the first channel smoothly as *x* increases. The heat transfer coefficient starts from the maximum observed value and decreases as *x* increases in the second channel. Again, similar trends were observed in Figure 36, Figure 37, Figure 38, Figure 41 and Figure 42. Figure 35, Figure 36, Figure 37 and Figure 38 indicate that the magnitude of the heat transfer coefficient in the first channel increases as Reynolds number increases. For example, at x/D = 48.2, the heat transfer coefficient increased by 156%, 382.19%, and 509.84%, as the Reynolds number increased from 100 to 200, 300, and 500, respectively. Similarly, at x/D = 177, the heat transfer coefficient increased by 140%, 440.49% and 725.78%, as Reynolds number increased from 100 to 200, 300, and 500, respectively. Similarly, the heat transfer coefficient increased with a Reynolds number in the case of water–Fe_3_O_4_ nanofluids (1 wt%), which can be seen in Figure 39, Figure 40, Figure 41 and Figure 42. For example, at x/D = 547, the heat transfer coefficient increased by 358.98%, 755.26%, and 844.81%, as the Reynolds number increased from 100 to 200, 300, and 500, respectively. Similarly, at x/D = 675, the heat transfer coefficient increased by 256.91%, 572.84% and 645.54%, as Reynolds number increased from 114 to 236, 347, and 524, respectively. 

The level of heat transfer coefficient in the second channel, compared to the first channel, increases as Reynolds number increases from 100 to 200, and then it starts decreasing when the Reynolds number increases from 300 to 500. In Figure 39, the magnitude of the heat transfer coefficient in the second channel is completely higher than the first channel. A similar, but milder, reduction in the heat transfer coefficient with Reynolds number was observed in the second channel when the working fluid was water–Fe_3_O_4_ nanofluids (1 wt%) (see Figure 39, Figure 40, Figure 41 and Figure 42). Most probably, the connector between the two microchannels increased the level of random motion of molecules and particles before entering the second channel. The level of enhancement of random motion is significant in low Reynolds numbers and becomes negligible as the Reynolds number increases. That is why the magnitude of the heat transfer coefficient increases in the second channel when the Reynolds number is relatively low, and it becomes insignificant at higher Reynolds numbers. Comparing Figure 35 and Figure 39, Figure 36 and Figure 40, Figure 37 and Figure 41, and Figure 38 and Figure 42, one can see that the level of heat transfer coefficient in the first channel and the second channel increases by adding Fe_3_O_4_ nanoparticles. For example, in the first channel when x/D = 177, the heat transfer coefficient increased by introducing Fe_3_O_4_ nanoparticles at 102.21%, 206.21%, 207.17%, and 254.38% when the Reynolds number was in range of 100 to 114, 200 to 236, 300 to 347, and 500 to 524, respectively. Obviously, adding Fe_3_O_4_ nanoparticles into deionized water increases the viscosity and thermal conductivity of nanofluids. As the viscosity of the nanofluid increases, the level of random motion of molecules and particles decreases which has a negative impact on the heat transfer coefficient, and, consequently, the heat transfer coefficient decreases. On the other hand, as the thermal conductivity of the nanofluid increases, the heat transfer coefficient increases. The overall impact of nanoparticles on the heat transfer coefficient depends on which impact is dominated. In the current case, the impact of nanoparticles on the thermal conductivity of nanofluids is dominated and, consequently, the heat transfer coefficient is enhanced. It was observed that adding Fe_3_O_4_ nanoparticles into deionized water increased the heat transfer coefficient in the first channel and the second channel as well.

## 9. Conclusions

In this paper, a brief literature review on the optimization and effects of nanofluids on the heat transfer was conducted, followed by an experimental study on the effects of a connector between two microchannels. The literature review indicated that introducing nanoparticles into the base liquid increased the viscosity and thermal conductivity of the working fluid in most cases. The random motion of molecules and nanoparticles would be suppressed by increasing the viscosity of the base liquid, which has a negative impact on the heat transfer coefficient. Simultaneously, adding nanoparticles with high thermal conductivity into the base liquid would increase the thermal conductivity of nanofluid and, consequently, it would increase the heat transfer coefficient. The overall impact of nanoparticles on the heat transfer coefficient depends on which effect is dominant. If the effect of thermal conductivity enhancement is dominated, the heat transfer coefficient increases. Otherwise, the heat transfer coefficient decreases. It was observed that adding Fe_3_O_4_ nanoparticles increased the overall thermal conductivity of the base liquid, and, consequently, the nanofluid heat transfer coefficient increased. It was also observed that as the temperature increases, nanofluid thermal conductivity increases and nanofluid viscosity decreases for given conditions.

The effects of the connector between two stainless-steel microchannels on the heat transfer coefficient of the second channel were investigated by changing the working fluid and Reynolds number. It was observed that the working fluid would refresh its memory inside the connector before entering the second channel; therefore, the heat transfer coefficient starts from a maximum value and decreases as *x* increases. As a result, the overall heat transfer coefficient of two channels increases by adding a connector between two microchannels. In addition, the connector increases the level of random motion of molecules and nanoparticles of the working fluid before entering the second channel, consequently increasing the heat transfer coefficient in the second microchannel. The enhancement of random motion of molecules and nanoparticles of the working fluid is significant when the Reynolds number is low, and becomes negligible as the Reynolds number increases. It was also observed that, in low Reynolds numbers, the heat transfer coefficient increases at the end of the second channel. In a low Reynolds number fluid flow, the fluid temperature increases at the end of the second channel. As a result, the viscosity of working fluid decreases; therefore, the random motion of molecules and particles increases and, consequently, the heat transfer coefficient increases. This phenomenon becomes weak as the Reynolds number increases further.

The effects of adding Fe_3_O_4_ nanoparticles into deionized water on the forced convection heat transfer coefficient inside the first and second stainless-steel microchannel were investigated. It was observed that deionized water–Fe_3_O_4_ nanofluid had higher heat transfer coefficients compared to deionized water, which is related to the higher thermal conductivity of the water–Fe_3_O_4_ nanofluid comparatively. In addition, the Fe_3_O_4_ nanoparticles increase the level of random motion and energy transfer from one layer to another.

The effects of the Reynolds number on the heat transfer coefficient inside the microchannel were investigated as well. In general, it was observed that the heat transfer coefficient increases as the Reynolds number increases, which is clearly related to the enhancement of random motion of molecules and particles as the Reynolds number increases.

## Figures and Tables

**Table 1:** Summary of nanofluid properties.

Material	Density [kg/m^3^]	Specific Heat [J/kg-K]	Dynamic Viscosity [mPa-s]	Thermal Conductivity [W/m-K]
Nanoparticles				
Fe_3_O_4_	5810	670	-	-
Nanofluid				
Fe_3_O_4_–water ^1^	997.9	4173	0.4889	0.7155
Water	988.0	4179.6	0.5471	0.6371

^1^ For $T_{ave}=45.54$ °C.

## Data Availability

The data presented in this study are available on request from the corresponding author.

## References

[1] Choi S.U.S., Eastman J.A. Enhancing Thermal Conductivity of Fluids with Nanoparticles. Proceedings of the International Mechanical Engineering Congress and Exhibition.

[2] Minea A.A., Moldoveanu M.G. (2017). Studies on Al_2_O_3_, CuO, and TiO_2_ water-based nanofluids: A comparative approach in laminar and turbulent flow. J. Eng. Thermophys..

[3] Chabi A.R., Zarrinabadi S., Peyghambarzadeh S.M., Hashemabadi S.H., Salimi M. (2017). Local convective heat transfer coefficient and friction factor of CuO/water nanofluid in a microchannel heat sink. Heat Mass Transf..

[4] He Y., Jin Y., Chen H., Ding Y., Cang D., Lu H. (2007). Heat transfer and flow behaviour of aqueous suspensions of TiO_2_ nanoparticles (nanofluids) flowing upward through a vertical pipe. Int. J. Heat Mass Transf..

[5] Sharma K.V., Sundar L.S., Sarma P.K. (2009). Estimation of heat transfer coefficient and friction factor in the transition flow with low volume concentration of Al_2_O_3_ nanofluid flowing in a circular tube and with twisted tape insert. Int. Commun. Heat Mass Transf..

[6] Hussien A.A., Abdullah M.Z., Al-Nimr M.A. (2016). Single-phase heat transfer enhancement in micro/minichannels using nanofluids: Theory and applications. Appl. Energy.

[7] Moreira T.A., Moreira D.C., Ribatski G. (2018). Nanofluids for heat transfer applications: A review. J. Braz. Soc. Mech. Sci. Eng..

[8] Chiam H.W., Azmi W.H., Usri N.A., Mamat R., Adam N.M. (2017). Thermal conductivity and viscosity of Al_2_O_3_ nanofluids for different based ratio of water and ethylene glycol mixture. Exp. Therm. Fluid Sci..

[9] Pryazhnikov M.I., Minakov A.V., Rudyak V.Y., Guzei D.V. (2017). Thermal conductivity measurements of nanofluids. Int. J. Heat Mass Transf..

[10] Khairul M., Shah K., Doroodchi E., Azizian R., Moghtaderi B. (2016). Effects of surfactant on stability and thermo-physical properties of metal oxide nanofluids. Int. J. Heat Mass Transf..

[11] Yu W., Xie H., Li Y., Chen L. (2011). Experimental investigation on thermal conductivity and viscosity of aluminum nitride nanofluid. Particuology.

[12] Lee J., Hwang K.S., Jang S.P., Lee B.H., Kim J.H., Choi S.U.S., Choi C.J. (2008). Effective viscosities and thermal conductivities of aqueous nanofluids containing low volume concentrations of Al_2_O_3_ nanoparticles. Int. J. Heat Mass Transf..

[13] Nadooshan A.A., Eshgarf H., Afrand M. (2018). Measuring the viscosity of Fe_3_O_4_-MWCNTs/EG hybrid nanofluid for evaluation of thermal efficiency: Newtonian and non-Newtonian behavior. J. Mol. Liq..

[14] Garoosi F. (2020). Presenting two new empirical models for calculating the effective dynamic viscosity and thermal conductivity of nanofluids. Powder Technol..

[15] Sundar L.S., Singh M.K., Sousa A.C.M. (2016). Experimental thermal conductivity and viscosity of nanodiamond-based propylene glycol and water mixtures. Diam. Relat. Mater..

[16] Akhavan-Zanjani H., Saffar-Avval M., Mansourkiaei M., Sharif F., Ahadi M. (2016). Experimental investigation of laminar forced convective heat transfer of Graphene–water nanofluid inside a circular tube. Int. J. Therm. Sci..

[17] Godson L., Raja B., Lal D.M., Wongwises S. (2010). Enhancement of heat transfer using nanofluids—An overview. Renew. Sustain. Energy Rev..

[18] Xie H., Li Y., Yu W. (2010). Intriguingly high convective heat transfer enhancement of nanofluid coolants in laminar flows. Phys. Lett. A.

[19] Patel H.E., Sundararajan T., Das S.K. (2010). An experimental investigation into the thermal conductivity enhancement in oxide and metallic nanofluids. J. Nanoparticle Res..

[20] Sulgani M.T., Karimipour A. (2019). Improve the thermal conductivity of 10w40-engine oil at various temperature by addition of Al_2_O_3_/Fe2O3 nanoparticles. J. Mol. Liq..

[21] Sundar L.S., Ramana E.V., Singh M.K., Sousa A.C.M. (2014). Thermal conductivity and viscosity of stabilized ethylene glycol and water mixture Al_2_O_3_ nanofluids for heat transfer applications: An experimental study. Int. Commun. Heat Mass Transf..

[22] Chon C.H., Kihm K.D. (2005). Empirical correlation finding the role of temperature and particle size for nanofluid (Al_2_O_3_) thermal conductivity enhancement. Appl. Phys. Lett..

[23] Pastoriza-Gallego M.J., Lugo L., Legido J.L., Piñeiro M.M. (2011). Thermal conductivity and viscosity measurements of ethylene glycol-based Al_2_O_3_ nanofluids. Nanoscale Res. Lett..

[24] Gangadevi R., Vinayagam B.K. (2019). Experimental determination of thermal conductivity and viscosity of different nanofluids and its effect on a hybrid solar collector. J. Therm. Anal. Calorim..

[25] Xing M., Yu J., Wang R. (2015). Experimental study on the thermal conductivity enhancement of water based nanofluids using different types of carbon nanotubes. Int. J. Heat Mass Transf..

[26] Sridhar S.V., Karuppasamy R., Sivakumar G.D. (2020). Experimental Investigation of Heat Transfer Enhancement of Shell and Tube Heat Exchanger Using SnO_2_-Water and Ag-Water Nanofluids. J. Therm. Sci. Eng. Appl..

[27] Singh K., Kumar S. (2020). An Experimental Study on Characterization of CuO/Water Nanofluid. Int. Res. J. Eng. Technol..

[28] Turgut A., Tavman I., Chirtoc M., Schuchmann H.P., Sauter C., Tavman S. (2009). Thermal Conductivity and Viscosity Measurements of Water-Based TiO_2_ Nanofluids. Int. J. Thermophys..

[29] Sundar L.S., Hortiguela M.J., Singh M.K., Sousa A.C.M. (2016). Thermal conductivity and viscosity of water based nanodiamond (ND) nanofluids: An experimental study. Int. Commun. Heat Mass Transf..

[30] Lodhi M.S., Sheorey T., Dutta G. (2020). Single-phase fluid flow and heat transfer characteristics of nanofluid in a circular microchannel: Development of flow and heat transfer correlations. J. Mech. Eng. Sci..

[31] Afrand M., Toghraie D., Sina N. (2016). Experimental study on thermal conductivity of water-based Fe_3_O_4_ nanofluid: Development of a new correlation and modeled by artificial neural network. Int. Commun. Heat Mass Transf..

[32] Yeganeh M., Shahtahmasebi N., Kompany A., Goharshadi E.K., Youssefi A., Siller L. (2010). Volume fraction and temperature variations of the effective thermal conductivity of nanodiamond fluids in deionized water. Int. J. Heat Mass Transf..

[33] Sundar L.S., Singh M.K., Sousa A.C.M. (2013). Investigation of thermal conductivity and viscosity of Fe_3_O_4_ nanofluid for heat transfer applications. Int. Commun. Heat Mass Transf..

[34] Godson L., Raja B., Lal D.M., Wongwises S. (2010). Experimental Investigation on the Thermal Conductivity and Viscosity of Silver-Deionized Water Nanofluid. J. Therm. Energy Gener. Transp. Storage Convers..

[35] Esfe M.H., Afrand M., Yan W., Akbari M. (2015). Applicability of artificial neural network and nonlinear regression to predict thermal conductivity modeling of Al_2_O_3_–water nanofluids using experimental data. Int. Commun. Heat Mass Transf..

[36] Maheshwary P.B., Handa C.C., Nemade K.R. (2017). A comprehensive study of effect of concentration, particle size and particle shape on thermal conductivity of titania/water based nanofluid. Appl. Therm. Eng..

[37] Main K., Eberl B., McDaniel D., Tikadar A., Paul T.C., Khan J.A. Nanoparticles shape effect on viscosity and thermal conductivity of ionic liquids based nanofluids. Proceedings of the 5th Thermal and Fluids Engineering Conference (TFEC).

[38] Zhu D., Wang L., Yu W., Xie H. (2018). Intriguingly high thermal conductivity increment for CuO nanowires contained nanofluids with low viscosity. Sci. Rep..

[39] Omrani A.N., Esmaeilzadeh E., Jafari M., Behzadmehr A. (2019). Effects of multi walled carbon nanotubes shape and size on thermal conductivity and viscosity of nanofluids. Diam. Relat. Mater..

[40] Kwek D., Crivoi A., Duan F. (2010). Effects of Temperature and Particle Size on the Thermal Property Measurements of Al_2_O_3_−Water Nanofluids. J. Chem. Eng. Data.

[41] Das P.K., Islam N., Santra A.K., Ganguly R. (2017). Experimental investigation of thermophysical properties of Al_2_O_3_–water nanofluid: Role of surfactants. J. Mol. Liq..

[42] Das P.K., Mallik A.K., Ganguly R., Santra A.K. (2018). Stability and thermophysical measurements of TiO_2_ (anatase) nanofluids with different surfactants. J. Mol. Liq..

[43] Freitas S.S., Silveria V., Jabardo J.M.S., Arce A.C. (2020). MWCNT and COOH–MWCNT aqueous nanofluids: Thermophysical and rheological characterization. J. Braz. Soc. Mech. Sci. Eng..

[44] Bouguerra N., Khabou A., Poncet S., Elkoun S. (2016). Thermal Conductivity of Al_2_O_3_/Water-Based Nanofluids: Revisiting the Influences of pH and Surfactant. Int. J. Mech. Aerosp. Ind. Mechatron. Manuf. Eng..

[45] Li X.F., Zhu D.S., Wang X.J., Wang N., Gao J.W., Li H. (2008). Thermal conductivity enhancement dependent pH and chemical surfactant for Cu-H_2_O nanofluids. Thermochim. Acta.

[46] Animasaun I.L., Yook S., Muhammad T., Mathew A. (2022). Dynamics of ternary-hybrid nanofluid subject to magnetic flux density and heat source or sink on a convectively heated surface. Surf. Interfaces.

[47] Adun H., Kavaz D., Dagbasi M. (2021). Review of ternary hybrid nanofluid: Synthesis, stability, thermophysical properties, heat transfer applications, and environmental effects. J. Clean. Prod..

[48] Elnaqeeb T., Animasaun I.L., Shah N.A. (2021). Ternary-hybrid nanofluids: Significance of suction and dual-stretching on three-dimensional flow of water conveying nanoparticles with various shapes and densities. Z. Nat. A.

[49] Mousavi S.M., Esmaeilzadeh F., Wang X.P. (2019). Effects of temperature and particles volume concentration on the thermophysical properties and the rheological behavior of CuO/MgO/TiO_2_ aqueous ternary hybrid nanofluid. J. Therm. Anal. Calorim..

[50] Nabil M.F., Azmi W.H., Hamid K.A., Mamat R., Hagos F.Y. (2017). An experimental study on the thermal conductivity and dynamic viscosity of TiO_2_-SiO_2_ nanofluids in water: Ethylene glycol mixture. Int. Commun. Heat Mass Transf..

[51] Wang F., Han L., Zhang Z., Fang X., Shi J., Ma W. (2012). Surfactant-free ionic liquid-based nanofluids with remarkable thermal conductivity enhancement at very low loading of graphene. Nanoscale Res. Lett..

[52] Al-Waeli A.H.A., Chaichan M.T., Sopian K., Kazem H.A. (2019). Influence of the base fluid on the thermo-physical properties of PV/T nanofluids with surfactant. Case Stud. Therm. Eng..

[53] Kumar V., Sahoo R.R. (2019). Viscosity and thermal conductivity comparative study for hybrid nanofluid in binary base fluids. Heat Transfer.

[54] Sundar L.S., Ramana E.V., Singh M., Sousa A.D. (2012). Viscosity of low volume concentrations of magnetic Fe_3_O_4_ nanoparticles dispersed in ethylene glycol and water mixture. Chem. Phys. Lett..

[55] Sundar L.S., Ramana E.V., Singh M.K., Gracio J., Sousa A.C.M. (2014). Preparation, Thermal and Rheological Properties of Propylene Glycol and Water Mixture Based Fe_3_O_4_ Nanofluids. J. Nanofluids.

[56] Yiamsawas T., Dalkilic A.S., Mahian O., Wongwises S. (2013). Measurement and Correlation of the Viscosity of Water-Based Al_2_O_3_ and TiO_2_ Nanofluids in High Temperatures and Comparisons with Literature Reports. J. Dispers. Sci. Technol..

[57] Vandrangi S.K., Hassan S., Sharma K.V., Akilu S., Emani S., Nabipour N. (2020). Effect of base fluids on thermo-physical properties of SiO_2_ nanofluids and development of new correlations. Math. Methods Appl. Sci..

[58] Gao D., Bai M., Hu C., Lv J., Wang C., Zhang X. (2020). Investigating control of convective heat transfer and flow resistance of Fe_3_O_4_/deionized water nanofluid in magnetic field in laminar flow. Nanotechnology.

[59] Nguyen C.T., Desgranges F., Roy G., Galanis N., Maré T., Boucher S., Mintsa H.A. (2007). Temperature and particle-size dependent viscosity data for water-based nanofluids—Hysteresis phenomenon. Int. J. Heat Fluid Flow.

[60] Malekzadeh A., Pouranfard A.R., Hatami N., Banari A.K., Rahimi M.R. (2016). Experimental Investigations on the Viscosity of Magnetic Nanofluids under the Influence of Temperature, Volume Fractions of Nanoparticles and External Magnetic Field. J. Appl. Fluid Mech..

[61] Jia-Fei Z., Zhong-Yang L., Ming-Jiang N., Ke-Fa C. (2009). Dependence of Nanofluid Viscosity on Particle Size and pH Value. Chin. Phys. Lett..

[62] Rudyak V.Y., Minakov A.V. (2018). Thermophysical properties of nanofluids. Eur. Phys. J. E.

[63] Bidgoli M.R., Kolahchi R., Karimi M.S. (2016). An experimental study and new correlations of viscosity of ethylene glycol-water based nanofluid at various temperatures and different solid concentrations. Struct. Eng. Mech..

[64] Abdul Hamid K., Azmi W., Mamat R., Usri N., Najafi G. (2015). Investigation of Al_2_O_3_ Nanofluid Viscosity for Different Water/EG Mixture Based. Energy Procedia.

[65] Ahmed W., Kazi S.N., Chowdhury Z.Z., Johan M.R.B., Mehmood S., Soudagar M.E.M., Mujtaba M.A., Gul M., Ahmad M.S. (2021). Heat transfer growth of sonochemically synthesized novel mixed metal oxide ZnO+Al_2_O_3_+TiO_2_/DW based ternary hybrid nanofluids in a square flow conduit. Renew. Sustain. Energy Rev..

[66] Sahoo R.R., Kumar V. (2020). Development of a new correlation to determine the viscosity of ternary hybrid nanofluid. Int. Commun. Heat Mass Transf..

[67] Sundar L.S., Singh M.K., Sousa A.C. (2013). Thermal conductivity of ethylene glycol and water mixture based Fe_3_O_4_ nanofluid. Int. Commun. Heat Mass Transf..

[68] Prasher R., Bhattacharya P., Phelan P.E. (2006). Brownian-Motion-Based Convective-Conductive Model for the Effective Thermal Conductivity of Nanofluids. J. Heat Transfer..

[69] Apmann K., Fulmer R., Soto A., Vafaei S. (2021). Thermal Conductivity and Viscosity: Review and Optimization of Effects of Nanoparticles. Materials.

[70] Bayat J., Nikseresht A.H. (2011). Investigation of the different base fluid effects on the nanofluids heat transfer and pressure drop. Heat Mass Transf..

[71] Maïga S.E.B., Palm S.J., Nguyen C.T., Roy G., Galanis N. (2005). Heat transfer enhancement by using nanofluids in forced convection flows. Int. J. Heat Fluid Flow.

[72] Aravind S.S.J., Ramaprabhu S. (2012). Graphene wrapped multiwalled carbon nanotubes dispersed nanofluids for heat transfer applications. J. Appl. Phys..

[73] Ebrahimnia-Bajestan E., Moghadam M.C., Niazmand H., Daungthongsuk W., Wongwises S. (2016). Experimental and numerical investigation of nanofluids heat transfer characteristics for application in solar heat exchangers. Int. J. Heat Mass Transf..

[74] Baby T.T., Ramaprabhu S. (2011). Synthesis and nanofluid application of silver nanoparticles decorated graphene. J. Mater. Chem..

[75] Aravind S.S.J., Baskar P., Baby T.T., Sabareesh R.K., Das S., Ramaprabhu S. (2011). Investigation of Structural Stability, Dispersion, Viscosity, and Conductive Heat Transfer Properties of Functionalized Carbon Nanotube Based Nanofluids. J. Phys. Chem. C.

[76] Sundararaj A.J., Pillai B.C., Asirvatham L.G. (2018). Convective heat transfer analysis of refined kerosene with alumina particles for rocketry application. J. Mech. Sci. Technol..

[77] Krishnakumar T.S., Sheeba A., Mahesh M., Prakash M.J. (2019). Heat transfer studies on ethylene glycol/water nanofluid containing TiO_2_ nanoparticles. Int. J. Refrig..

[78] Bhanvase B.A., Sarode M.R., Putterwar L.A., Abdullah K.A., Deosarkar M.P., Sonawane S.H. (2014). Intensification of convective heat transfer in water/ethylene glycol based nanofluids containing TiO_2_ nanoparticles. Chem. Eng. Process. Process Intensif..

[79] Mohan M., Thomas S., Sobhan C.B. (2020). Convective heat transfer studies in dilute alumina and silica nanofluids flowing through a channel using Mach-Zehnder interferometry. Heat Mass Transf..

[80] Utomo A.T., Poth H., Robbins P.T., Pacek A.W. (2012). Experimental and theoretical studies of thermal conductivity, viscosity and heat transfer coefficient of titania and alumina nanofluids. Int. J. Heat Mass Transf..

[81] Ding Y., Chen H., He Y., Lapkin A., Yeganeh M., Šiller L., Butenko Y.V. (2007). Forced convective heat transfer of nanofluids. Adv. Powder Technol..

[82] Heris S.Z., Farzin F., Sardarabadi H. (2015). Experimental Comparison Among Thermal Characteristics of Three Metal Oxide Nanoparticles/Turbine Oil-Based Nanofluids Under Laminar Flow Regime. Int. J. Thermophys..

[83] Vajjha R.S., Das D.K., Namburu P.K. (2010). Numerical study of fluid dynamic and heat transfer performance of Al_2_O_3_ and CuO nanofluids in the flat tubes of a radiator. Int. J. Heat Fluid Flow.

[84] Pattanayak B., Mund A., Jayakumar J.S., Parashar K., Parashar S.K.S. (2020). Estimation of Nusselt number and effectiveness of double-pipe heat exchanger with Al_2_O_3_–, CuO–, TiO_2_–, and ZnO–water based nanofluids. Heat Transf..

[85] Khairul M.A., Doroodchi E., Azizian R., Moghtaderi B. (2017). The influence of different flow regimes on heat transfer performance and exergy loss of Al_2_O_3_/DI-water and CuO/DI-water nanofluids. Appl. Therm. Eng..

[86] Hussien A.A., Abdullah M.Z., Yusop N.M., Al-Nimr M.A., Atieh M.A., Mehrali M. (2017). Experiment on forced convective heat transfer enhancement using MWCNTs/GNPs hybrid nanofluid and mini-tube. Int. J. Heat Mass Transf..

[87] Zamzamian A., Oskouie S.N., Doosthoseini A., Joneidi A., Pazouki M. (2011). Experimental investigation of forced convective heat transfer coefficient in nanofluids of Al_2_O_3_/EG and CuO/EG in a double pipe and plate heat exchangers under turbulent flow. Exp. Therm. Fluid Sci..

[88] Esmaeilzadeh E., Almohammadi H., Vatan S.N., Omrani A.N. (2013). Experimental investigation of hydrodynamics and heat transfer characteristics of γ-Al_2_O_3_/water under laminar flow inside a horizontal tube. Int. J. Therm. Sci..

[89] Hwang K.S., Jang S.P., Choi S.U.S. (2009). Flow and convective heat transfer characteristics of water-based Al_2_O_3_ nanofluids in fully developed laminar flow regime. Int. J. Heat Mass Transf..

[90] Ali H.M. (2020). In tube convection heat transfer enhancement: SiO_2_ aqua based nanofluids. J. Mol. Liq..

[91] Rea U., McKrell T., Hu L., Buongiorno J. (2009). Laminar convective heat transfer and viscous pressure loss of alumina–water and zirconia–water nanofluids. Int. J. Heat Mass Transf..

[92] Kai L.C., Abdullah M.Z., Ismail M.A., Mamat H. (2019). Enhancement of nanofluid heat transfer in a mini-tube using SiO_2_ nanoparticles. Adv. Mater. Process. Technol..

[93] Umer A., Naveed S., Ramzan N. (2016). Experimental study of laminar forced convective heat transfer of deionized water based copper (I) oxide nanofluids in a tube with constant wall heat flux. Heat Mass Transf..

[94] Minakov A.V., Lobasov A.S., Guzei D.V., Pryazhnikov M.I., Rudyak V.Y. (2015). The experimental and theoretical study of laminar forced convection of nanofluids in the round channel. Appl. Therm. Eng..

[95] Wen D., Ding Y. (2004). Experimental investigation into convective heat transfer of nanofluids at the entrance region under laminar flow conditions. Int. J. Heat Mass Transf..

[96] Elias M., Miqdad M., Mahbubul I., Saidur R., Kamalisarvestani M., Sohel M., Hepbasli A., Rahim N., Amalina M. (2013). Effect of nanoparticle shape on the heat transfer and thermodynamic performance of a shell and tube heat exchanger. Int. Commun. Heat Mass Transf..

[97] Elias M.M., Shahrul I.M., Mahbubul I.M., Saidur R., Rahim N.A. (2014). Effect of different nanoparticle shapes on shell and tube heat exchanger using different baffle angles and operated with nanofluid. Int. J. Heat Mass Transf..

[98] Chen H., Yang W., He Y., Ding Y., Zhang L., Tan C., Lapkin A.A., Bavykin D.V. (2008). Heat transfer and flow behaviour of aqueous suspensions of titanate nanotubes (nanofluids). Powder Technol..

[99] Maheshwary P.B., Handa C.C., Nemade K.R., Chaudhary S.R. (2020). Role of nanoparticle shape in enhancing the thermal conductivity of nanofluids. Mater. Today: Proc..

[100] Ekiciler R., Çetinkaya M.S.A., Arslan K. (2020). Heat transfer enhancement in an equilateral triangular duct by using an Al_2_O_3_/water nanofluid: Effect of nanoparticle shape and volume fraction. Heat Transf. Res..

[101] Norouzipour A., Abdollahi A., Afrand M. (2019). Experimental study of the optimum size of silica nanoparticles on the pool boiling heat transfer coefficient of silicon oxide/deionized water nanofluid. Powder Technol..

[102] Timofeeva E.V., Yu W., France D.M., Singh D., Routbort J.L. (2010). Base fluid and temperature effects on the heat transfer characteristics of SiC in ethylene glycol/H_2_O and H_2_O nanofluids. J. Appl. Phys..

[103] Anoop K.B., Sundararajan T., Das S.K. (2009). Effect of particle size on the convective heat transfer in nanofluid in the developing region. Int. J. Heat Mass Transf..

[104] Heidarshenas A., Azizi Z., Peyghambarzadeh S.M., Sayyahi S. (2020). Experimental investigation of the particle size effect on heat transfer coefficient of Al_2_O_3_ nanofluid in a cylindrical microchannel heat sink. J. Therm. Anal. Calorim..

[105] Zhang L., Zhang A., Jing Y., Qu P., Wu Z. (2021). Effect of Particle Size on the Heat Transfer Performance of SiO_2_–Water Nanofluids. J. Phys. Chem. C.

[106] Murshed S.M.S., Castro C.A.N., Ahsan A. (2011). Forced Convective Heat Transfer of Nanofluids in Minichannels. Two Phase Flow, Phase Change and Numerical Modeling.

[107] Xuan Y., Li Q., Tie P. (2013). The effect of surfactants on heat transfer feature of nanofluids. Exp. Therm. Fluid Sci..

[108] Halefadl S., Estellé P., Maré T. (2014). Heat transfer properties of aqueous carbon nanotubes nanofluids in coaxial heat exchanger under laminar regime. Exp. Therm. Fluid Sci..

[109] Ding Y., Alias H., Wen D., Williams R.A. (2006). Heat transfer of aqueous suspensions of carbon nanotubes (CNT nanofluids). Int. J. Heat Mass Transf..

[110] Hosseinipour E., Heris S.Z., Shanbedi M. (2016). Experimental investigation of pressure drop and heat transfer performance of amino acid-functionalized MWCNT in the circular tube. J. Therm. Anal. Calorim..

[111] Mukherjee S., Jana S., Mishra P.C., Chaudhuri P., Chakrabarty S. (2021). Experimental investigation on thermo-physical properties and subcooled flow boiling performance of Al_2_O_3_/water nanofluids in a horizontal tube. Int. J. Therm. Sci..

[112] Kim D., Kwon Y., Cho Y., Li C., Cheong S., Hwang Y., Lee J., Hong D., Moon S. (2009). Convective heat transfer characteristics of nanofluids under laminar and turbulent flow conditions. Curr. Appl. Phys..

[113] Leong K.Y., Saidur R., Kazi S.N., Mamun A.H. (2010). Performance investigation of an automotive car radiator operated with nanofluid-based coolants (nanofluid as a coolant in a radiator). Appl. Therm. Eng..

[114] Liu D., Yu L. (2011). Single-Phase Thermal Transport of Nanofluids in a Minichannel. J. Heat Transfer..

[115] Nguyen C.T., Roy G., Gauthier C., Galanis N. (2007). Heat transfer enhancement using Al_2_O_3_–water nanofluid for an electronic liquid cooling system. Appl. Therm. Eng..

[116] Gupta M., Singh V., Kumar S., Dilbaghi N. (2020). Experimental analysis of heat transfer behavior of silver, MWCNT and hybrid (silver +MWCNT) nanofluids in a laminar tubular flow. J. Therm. Anal. Calorim..

[117] Cabaleiro D., Colla L., Agresti F., Lugo L., Fedele L. (2015). Transport properties and heat transfer coefficients of ZnO/(ethylene glycol + water) nanofluids. Int. J. Heat Mass Transf..

[118] Sabir R., Ramzan N., Umer A., Muryam H. (2015). An experimental study of forced convective heat transfer characteristic of gold water nanofluid in laminar flow. Sci. Int..

[119] Baby T.T., Sundara R. (2011). Synthesis and Transport Properties of Metal Oxide Decorated Graphene Dispersed Nanofluids. J. Phys. Chem. C.

[120] Ahmed W., Kazi S.N., Chowdhury Z.Z., Johan M.R.B., Akram N., Mujtaba M.A., Gul M., Oon C.S. (2021). Experimental investigation of convective heat transfer growth on ZnO@TiO_2_/DW binary composites/hybrid nanofluids in a circular heat exchanger. J. Therm. Anal. Calorim..

[121] Barai D.P., Bhanvase B.A., Saharan V.K. (2019). Reduced Graphene Oxide-Fe_3_O_4_ Nanocomposite Based Nanofluids: Study on Ultrasonic Assisted Synthesis, Thermal Conductivity, Rheology, and Convective Heat Transfer. Ind. Eng. Chem. Res..

[122] Haghighi E.B., Saleemi M., Nikkam N., Khodabandeh R., Toprak M.S., Muhammed M., Palm B. (2014). Accurate basis of comparison for convective heat transfer in nanofluids. Int. Commun. Heat Mass Transf..

[123] Qiang L., Yimin X. (2002). Convective heat transfer and flow characteristics of Cu-water nanofluid. Sci. China Ser. E: Technol. Sci..

[124] Kumaresan V., Khader S.M.A., Karthikeyan S., Velraj R. (2013). Convective heat transfer characteristics of CNT nanofluids in a tubular heat exchanger of various lengths for energy efficient cooling/heating system. Int. J. Heat Mass Transf..

[125] Jung J., Oh H., Kwak H. (2006). Forced Convective Heat Transfer of Nanofluids in Microchannels. ASME Int. Mech. Eng. Congr. Expo..

[126] Kim S., Tserengombo B., Choi S., Noh J., Huh S., Choi B., Chung H., Kim J., Jeong H. (2019). Experimental investigation of heat transfer coefficient with Al_2_O_3_ nanofluid in small diameter tubes. Appl. Therm. Eng..

[127] Heris S.Z., Esfahany M.N., Etemad S.G. (2007). Experimental investigation of convective heat transfer of Al_2_O_3_/water nanofluid in circular tube. Int. J. Heat Fluid Flow.

[128] Gnielinski V., Kabelac S., Kind M., Martin H., Mewes D., Schaber K., Stephan P. (2006). VDI-Wärmeatlas.

[129] Shah R.K., London A.L. (1978). Laminar Flow Forced Convection in Ducts A Source Book for Compact Heat Exchanger Analytical Data.

[130] Pourfayaz F., Sanjarian N., Kasaeian A., Astaraej F.R., Sameti M., Nasirivatan S. (2018). An experimental comparison of SiO_2_/water nanofluid heat transfer in square and circular cross-sectional channels. J. Therm. Anal. Calorim..

[131] Vajjha R.S., Das D.K. (2009). Specific Heat Measurement of Three Nanofluids and Development of New Correlations. J. Heat Transf..

[132] Vanapalli S., Brake H.J.M. (2013). Assessment of thermal conductivity, viscosity and specific heat of nanofluids in single phase laminar internal forced convection. Int. J. Heat Mass Transf..

[133] Bergman T.L. (2009). Effect of reduced specific heats of nanofluids on single phase, laminar internal forced convection. Int. J. Heat Mass Transf..

[134] Asadi A. (2018). A guideline towards easing the decision-making process in selecting an effective nanofluid as a heat transfer fluid. Energy Convers. Manag..

[135] Vajjha R.S., Das D.K., Kulkarni D.P. (2010). Development of new correlations for convective heat transfer and friction factor in turbulent regime for nanofluids. Int. J. Heat Mass Transf..

